# Hybrid mass spectrometry methods reveal lot-to-lot differences and delineate the effects of glycosylation on the tertiary structure of Herceptin®†
†Electronic supplementary information (ESI) available: Supporting results (Fig. S1–S23 and Tables S1–S3) as mentioned in the text. See DOI: 10.1039/c8sc05029e


**DOI:** 10.1039/c8sc05029e

**Published:** 2019-01-17

**Authors:** Rosie Upton, Lukasz G. Migas, Kamila J. Pacholarz, Richard G. Beniston, Sian Estdale, David Firth, Perdita E. Barran

**Affiliations:** a Manchester Institute of Biotechnology , Michael Barber Centre for Collaborative Mass Spectrometry , University of Manchester , 131 Princess Street , Manchester , M1 7DN , UK . Email: Perdita.barran@manchester.ac.uk; b Covance Laboratories Ltd. , Otley Road , Harrogate , HG3 1PY , UK

## Abstract

To quantify the measurable variations in the structure of a biopharmaceutical product we systematically evaluate three lots of Herceptin®, two mAb standards and an intact Fc-hinge fragment. Each mAb is examined in three states; glycan intact, truncated (following endoS2 treatment) and fully deglycosylated. Despite equivalence at the intact protein level, each lot of Herceptin® gives a distinctive signature in three different mass spectrometry approaches. Ion mobility mass spectrometry (IM-MS) shows that in the API, the attached N-glycans reduce the conformational spread of each mAb by 10.5–25%. Hydrogen/deuterium exchange mass spectrometry (HDX-MS) data support this, with lower global deuterium uptake in solution when comparing intact to the fully deglycosylated protein. HDX-MS and activated IM-MS map the influence of glycans on the mAb and reveal allosteric effects which extend far beyond the Fc domains into the Fab region. Taken together, these findings and the supplied interactive data sets establish acceptance criteria with application for MS based characterisation of biosimilars and novel therapeutic mAbs.

## Abstract 2

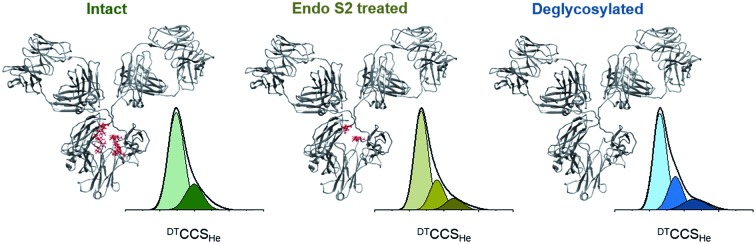
To quantify the measurable structural heterogeneity of a biopharmaceutical product and the effect of glycosylation on this we systematically evaluate three lots of Herceptin®, two mAb standards and an intact 5 Fc-hinge fragment.

## Introduction

As many as 70 monoclonal antibody (mAb) products are predicted to be on the market by 2020.^1^ To date, the majority of commercial mAb products are expressed in mammalian cell lines^2^ which are responsible for transcribing post-translational modifications (PTMs) such as glycosylation. Subtle differences in the cell cultures and manufacturing conditions including pH and temperature can lead to variations in the glycan profiles and structure of the mAbs.^3^ This process-related variability can therefore lead to changes in the biological activity and pharmacokinetics.

As mAb products come off patent, the opportunity to develop biosimilar candidates arises however, because mAb biologics are large (∼150 kDa), flexible molecules that can exhibit intrinsic heterogeneity through PTMs, truncations, as well as global features such as fold and aggregation propensity,^4^ the approval pathway for any new mAb or biosimilar is fraught with difficulty. Although guidelines are in place from the European Medicines Agency (EMA),^5^,^6^ the United States Food and Drug Administration (FDA)^7^,^8^ and the World Health Organisation (WHO),^9^ equivalence acceptance criteria currently need to be considered on a case-by-case basis for each mAb.^10^ With this in mind, thorough characterisation of any innovator therapeutic is required to understand the effects of primary and higher order structure, PTMs and biological activity upon function, efficacy and safety. One critical assessment to make, with direct relevance to the development and licencing of new biopharmaceuticals as well as to biosimilar production, is a comparison between lots of a given mAb product.

N-linked glycosylation of the conserved Fc asparagine residue (“Asn-297”) in mAb-therapeutics plays a key role in the immunological activity,^11^–^13^ structural conformation^14^,^15^ and overall stability^16^,^17^ of the IgG1 protein. Furthermore, since we have demonstrated lot-to-lot variability in the glycosylation profile of Herceptin®, it follows that measuring how glycosylation impacts the overall mAb conformation is of prime importance. Well established analytical techniques such as differential scanning calorimetry (DSC) and size exclusion chromatography (SEC) are routinely used within the biopharmaceutical industry to assess the thermal stability^18^,^19^ and aggregation properties^20^,^21^ of antibodies, respectively. In more recent years, mass spectrometry based techniques have been widely used for the characterisation of IgGs,^22^–^24^ quantification of glycosylation profiles^25^–^27^ and to study conformational changes that occur upon deglycosylation.^28^,^29^ Hydrogen–deuterium exchange mass spectrometry (HDX-MS) and ion mobility mass spectrometry (IM-MS) have provided new insights to mAb structure, providing rapid analysis from only a few micrograms of sample, and the former enabling structural differences to be mapped to regions of the protein fold.^29^–^32^

**Fig. 1:**
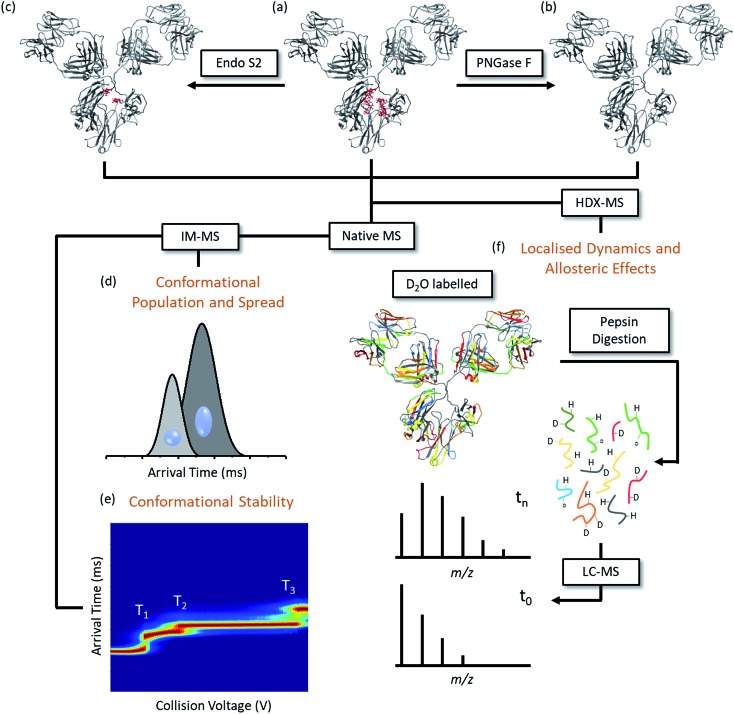
Experimental workflow summarising the methods utilized in this paper in order to assess the conformational spread and populations (d), the conformational stability (e) and the localised dynamics and allosteric effects (f) on Herceptin® between lots and as a function of glycosylation. Intact IgG1s (a) were digested with endoS2 (b) or PNGase F (c) to truncate or remove the N-linked glycosylation, respectively. The three mAb forms were analysed using ion mobility mass spectrometry and hydrogen/deuterium exchange mass spectrometry. IM-MS investigated the conformational spread (d) of each mAb form in the gas phase and identified different conformational families. Ions of the same mass-to-charge can be separated based upon their size and shape; compact ions will traverse the mobility cell faster than more extended ions. The arrival time distribution is converted into a collision cross section distribution^36^ allowing a comparison of the conformational space occupied by each sample. Collisional activation of the mAbs followed by ion mobility (e) measured the stability profiles of each mAb form and defined the conformational transitions (T_1_, T_2_, T_3_) across the collision voltage range. HDX-MS measures the solvent accessibility of the mAb backbone (f) and therefore provides insight into the structural dynamics of the protein. The samples are incubated with deuterated buffer for different time points (*t*_0_ is the reference and therefore is not deuterated) before the exchange reaction is quenched and the mAb is digested using pepsin. Mass spectrometry measurements calculate the mass shift (level of deuterium uptake) with respect to time providing information regarding the dynamic properties of specific regions on the mAb structure.

We have investigated the hypotheses presented by Berkowitz *et al.* that MS is a ‘valuable tool’ for probing the three-dimensional higher order structure both for lot-to-lot comparisons and for studying the effects of PTMs upon conformation.^15^ We employ three mass spectrometry methods to compare the global conformation and dynamics for Herceptin®, in three separately manufactured lots and for each at differing levels of Fc-domain N-linked glycosylation (Fig. 1). We and others have previously demonstrated the use of an endoglycosidase, endoS2, to dissect between the two N-acetyl glucosamine residues of the chitobiose core, leaving only a single GlcNAc or [GlcNAc + Fucose] stub to aid afucosylation quantification^26^ and high-mannose quantification.^33^ By using endoS2 alongside PNGase F we can investigate the conformational changes and dynamics of IgG1 mAbs with three differing levels of glycosylation; fully intact, ‘truncated’ (endoS2 treated) and fully deglycosylated (PNGase F treated). We have used HDX-MS to study different lots of Herceptin®, along with a mAb supplied by Waters as a *mass check* standard and an intact IgG1 Fc-hinge fragment. We find that upon glycan removal and ‘truncation’ there is an increase in deuterium uptake, indicative of an increased number of solvent accessible exchangeable amide hydrogens. For IM-MS studies, both collision cross section distributions (CCSD) and collision activated IM-MS heat maps are presented for different lots of Herceptin®, the Fc-hinge fragment and also the NIST mAb reference material.^34^ The NIST mAb is a useful standard for the evaluation of lot-to-lot comparisons as it has been fully homogenised across multiple lots prior to its release.^35^ The glycosylation profiles and therefore the conformational properties of the reference material is predicted to be typically more varied than individual lots.

## Materials and methods

### Materials

Herceptin® antibody lots were from Roche–Genentech. NIST monoclonal antibody reference material 8671 was purchased from NIST and the IgG1 Fc-hinge fragment was supplied by UCB. EndoS2 (GlycINATOR® and immobilised GlycINATOR®) were purchased from Genovis AB. PNGase F and ethylenediaminetetraacetic acid (EDTA) were purchased from Promega. Ammonium acetate, K_2_HPO_4_ and KH_2_PO_4_ were purchased from Fisher Scientific. Iodoacetamide, guanidine hydrochloride, trifluoroacetic acid (TFA), and tris(2-carboxyethyl) phosphine hydrochloride (TCEP) were purchased from Sigma-Aldrich. Lysyl Endopeptidase® was purchased from Wako Chemicals and Tris buffer was purchased from Fluka. Purified water was produced in-house using a Milli-Q Advantage A10 system. LC-MS grade acetonitrile and formic acid were purchased from VWR Chemicals and Fluka Analytical, respectively. All samples were desalted using micro Bio-Spin 6 columns (Bio-Rad). To reduce aggregation (<2%), nESI IM-MS mAb samples were prepared at 1 mg ml^–1^ (∼5 μm) in 100 mM ammonium acetate, pH 6.80. For HDX-MS mAb samples were prepared at 3 mg ml^–1^ (10 mM phosphate, pH 7.00), diluted ∼30-fold during the incubation/quench stages resulting in a <10 μg injection size. Previously reported LC-MS analysis of IdeS digested Herceptin® lots calculated Fc/2 and F(ab′)2 fragment masses to be within 12 ppm and 41 ppm of the expected masses, respectively [expected masses 25 317 Da (average) and 97 628 Da, respectively].^26^

### Enzymatic digestions to selectively cleave glycans

mAb samples (50 mM ammonium bicarbonate, pH 7.8) were incubated with PNGase F at 37 °C for 20 h, at optimised IgG : enzyme ratios. For endoS2 treated mAb samples, 1 mg ml^–1^ IgG was incubated at 37 °C for 30 min with endoS2 for nESI-IM-MS analyses (Fig. S1 in ESI†). For HDX-MS experiments, endoS2 digestion was performed using Immobilised GlycINATOR® (endoS2) cartridges to prevent His-tag interaction with the pepsin column. Prior to nESI-IM-MS or HDX-MS analyses the samples were buffer exchanged into 100 mM ammonium acetate or 10 mM phosphate buffers, respectively. MS spectra to confirm the complete enzymatic digestion of Herceptin®, the NIST mAb and the IgG1 Fc-hinge fragment samples are provided in Fig. S2, S3 and S4 in the ESI,† respectively. For comparison, the deconvoluted glycan profiles for each of the Herceptin lots are provided in Fig. S5.†

### IM-MS analysis

IM-MS experiments were acquired using a modified Waters Synapt G2 with a 25.05 cm RF-confining linear drift cell^37^ filled with ∼2 Torr helium, 298 K. A multi-field approach (DV range 213–114 V, 20 V increments) was applied using WREnS and in-house software (ORIGAMI).^38^ For nESI, in-house pulled borosilicate capillary tips (Sutter P-1000 micropipette puller system; capillary ID: 0.9 mm, OD: 1.2 mm) were used with platinum wire inserted; capillary voltage 1.2–1.4 kV, cone 100 V and source temperature 40 °C. The arrival time distributions (ATDs) were converted to collision cross section distributions (CCSDs) for the most intense charge states, allowing visualisation of the conformational spread adopted by the samples in the gas-phase. Summation of these CCSD plots, (*i.e.* removing the effects of charge), generates global CCSD plots which provide an overview of the conformational diversity of the sample. The global CCSDs for the NIST mAb were fitted with the minimum number of Gaussians required for the cumulative fit based upon diffusion limitations. Each Gaussian was defined and fixed by the FWHM and apex position before being transposed onto the global CCSDs of the Herceptin® lots.

### HDX-MS analysis

The HDX-MS setup comprised of a Waters nano-Acquity UPLC system with ESI MS detection coupled to a LEAP Technologies dual-armed robot for sample preparation, incubation and inlet injection. A Waters Synapt G2Si mass spectrometer was operated in positive ion/resolution mode, with data acquired over *m*/*z* range 290–2500. The LC gradient was supplied at 40 μL min^–1^ flow and peptides were eluted over 16 min with 85% organic (mobile phases: A, water + 0.1% formic acid; and B, acetonitrile + 0.1% formic acid). Samples were incubated with deuterated labelling buffer (10 mM phosphate in D_2_O, pD 7.00) for time points ranging from 0 min to 8 h, before being quenched and reduced by quench buffer (0.5 M TCEP, 4 M guanidine hydrochloride, 100 mM phosphate, pH 2.30) at 1 °C for 30 s. On-line pepsin digestion (Waters Enzymate BEH Pepsin 2.1 × 30 mm) at 20 °C for 3 min preceded the analytical column held at 0 °C (Waters Acquity UPLC BEH C18 1.7 μm, 1.0 × 10 mm) for chromatographic peptide separation. Data was acquired using Waters MassLynx software v4.1, with the LEAP robot controlled by HDx Director 1.0.3.9. Data processing and analysis were carried out with Waters ProteinLynx Global Server 3.0.1 and Waters DynamX 3.0 software, respectively.

### Collision activated IM-MS analysis

Experiments were performed on a Waters Synapt G2S using nESI and trap-activated ion mobility; capillary voltage 1.2–1.3 kV, cone 100 V and source temperature 40 °C. The trap region was pressurised at ∼3.22 × 10^–2^ mbar argon gas and for mobility separation the travelling-wave cell was operated at ∼2.85 mbar (35 V wave height, 600 m s^–1^ wave velocity). The 24^+^ charge state (most intense) was mass selected using the quadrupole prior to the trap region to ensure that recorded IM data was a result of ion activation and not charge stripping. ORIGAMI,^38^ was used to automatically acquire data for collision energies 4–200 V in 2 V increments as well as for data processing.

### Disulphide bond mapping

100 μg of mAb was incubated for 20 minutes at 57 °C in denaturing buffer (6 M guanidine hydrochloride, 50 mM Tris and 5 mM EDTA, pH 8). Each sample was prepared in duplicate; to one replicate 2.5 μL iodoacetamide (300 mM in 200 mM sodium phosphate, pH 6.5) was added and to the other 2.5 μL of water was added. A further 100 μL of water was added to each sample to dilute the guanidine hydrochloride. Lys-C was added in a 1 : 100 ratio and incubated at 37 °C overnight. TFA was added to a final concentration of 1%. Samples were analysed *via* LC-MS using a Waters Acquity UPLC coupled to a Waters Vion mass spectrometer operated in positive ion/resolution mode. The chromatographic separation was achieved using a Waters Acquity BEH C18 Peptide column (2.1 × 100 mm, 1.7 μm) and an LC gradient supplied at 0.25 ml min^–1^, over 90 min up to 90% organic (mobile phases; water, acetonitrile and 1% TFA). Waters UNIFI™ 1.8 was used to generate a list of possible disulphide bond linkages.

### Circular dichroism

Far UV-CD analysis was performed using a Chirascan™ CD spectrometer. Data was recorded over a wavelength range of 190–260 nm with a 0.5 nm step size. 1 mg ml^–1^ samples in 100 mM ammonium acetate buffer (concentration measured precisely using a Thermo NanoDrop 2000) were added to a 0.1 mm cuvette and analysed at 25 °C for 12 minutes (time per point = 5 s). The data was converted from millidegrees (mdeg) to molar ellipticity ([*θ*]) to correct for deviations in concentration between samples.

### Interactive figures

A number of main text and ESI figures† presented in this article were recreated in an interactive format to enable in-depth interrogation of the presented results. The interactive figures were created using ORIGAMI^38^ and require usage of a modern internet browser and access to the internet.

## Results and discussion

### Demonstrating conformational variability between lots and as a function of glycosylation with native ion mobility mass spectrometry

**Fig. 2:**
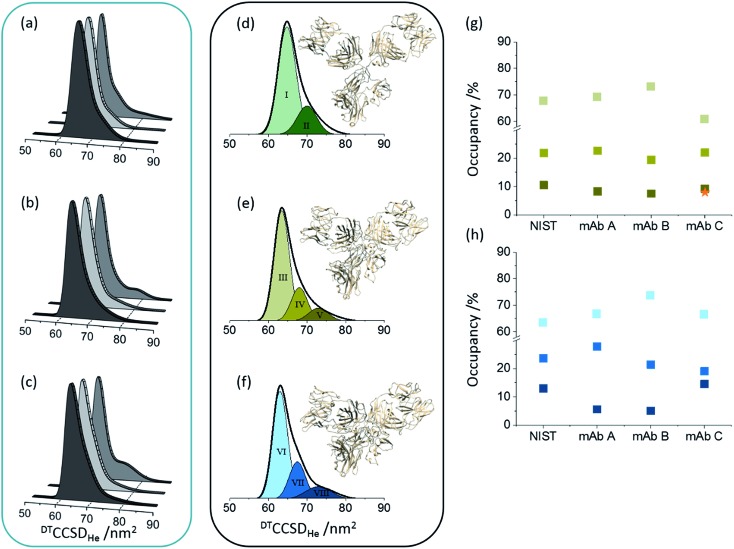
Global collision cross section distributions (^DT^CCSD_He_) for intact (a), endoS2 treated (b) and deglycosylated (c) Herceptin®; lot A (dark grey), lot B (light grey) and lot C (grey). Global CCSDs for intact (d), endoS2 treated (e) and deglycosylated (f) NIST mAb standard with fitted conformers. The darker the Gaussian for each sample, the more extended the conformer. The conformers present in each sample preparation have slightly different centres and FWHM values, hence different colours for each sample preparation. CCSDs for individual charge states for each sample and at each glycosylation level are provided in Fig. S7 in ESI.† Inserted mAb figures are snapshots from MD trajectories previously performed on pdb: ; 1IGY.^30^ The image in (d) is from early in the MD simulation and therefore represents the intact mAb; later snapshots represent more contracted forms (e and f). Percentage occupancies of each conformer in the endoS2 treated samples (g) and deglycosylated samples (h) are presented with colours corresponding to the fitted Gaussians. For the endoS2 treated Herceptin® lot C there is an additional fourth extended Gaussian (see Fig. S6 in ESI†) which is represented by the orange star. Standard deviations between three day-to-day replicates are provided for the ^DT^CCSD_He_ for individual charge states in Fig. S7 in ESI.†

Here we compared the distribution of conformers found for each Herceptin® mAb lot, and whilst broadly similar, each is distinctive (Fig. 2a). Drift time CCSD (^DT^CCSD_He_) plots show the highest similarity between Herceptin® lots A and B, whereas lot C possesses a comparably broader conformational profile. This analysis allows us to measure conformational lot-to-lot variability in the API. Intact mAbs are inherently heterogeneous consisting various glycoforms in many structural orientations. To mitigate this heterogeneity, enzymatic treatment can be used to dissect these glycan moieties resulting in a more homogenous molecule. Treating glycans with either PNGase F to deglycosylate or endoS2 to truncate (*i.e.* GlcNAc or [GlcNAc + Fucose] remaining) renders the CCS distributions more similar, yet notable conformational differences remain (Fig. 2b and c). The NIST mAb was analysed in the same way and presents a conformational spread most similar to those of Herceptin® lot C, indicating that the glycosylation in this lot is most variable and that this causes observable conformational differences.

Native mass spectrometry approaches have shown that proteins can retain a number of different conformations even in the absence of bulk solution, which can be related to solvated data.^39^,^40^ For the NIST mAb eight conformational familes were fitted across the intact, endoS2 treated and deglycosylated samples (Fig. 2d–f). The most compact conformer in the intact sample (I) contracts ∼3.5% (conformers III and VI) following glycan modification whilst introducing more extended conformers (V and VIII). Transposing these defined, Gaussian-fitted conformations onto the Herceptin® lots allows visualisation of the conformational space occupied, relative to a standard (Fig. S6 in ESI†). The distributions present in the NIST mAb align well with the global CCSDs measured for Herceptin® lots A and B, whereas Herceptin® lot C presents a notably more extended profile with the addition of a ninth conformational family occupied in the endoS2 treated sample (Fig. S6 in ESI†). Tracking the occupancy of each conformation across all endoS2 treated and deglycosylated samples allows the lots to be compared (Fig. 2g and h). In Fig. 2g the additional fourth conformer (orange star) for endoS2 treated Herceptin® lot C indicates destabilisation *cf.* the other samples. The mAb structures in Fig. 2d–f illustrate the conformational collapse which is observed following glycan truncation or removal. These have been adapted from previously reported molecular dynamic (MD) simulations of an IgG1 which confirmed that despite a conformational collapse, secondary structure is largely retained.^30^

CCSD plots, both at the local and global level, were also produced for an IgG1 Fc-hinge fragment (ESI Fig. S8†) to detail conformational changes in the C_H_2 and C_H_3 domains of IgG1s. The data supports the observations made in the full mAb IM-MS analyses (Fig. 2 and S6†) where flexibility is enumerated as the width of the CCSDs. Taken together both the Fc-hinge fragment and the full IgG1s change in structure as a function of glycosylation in the following order intact < deglycosylated < endoS2 treated.

### Intrinsic flexibility of mAbs is revealed by collisional activation followed by ion mobility mass spectrometry

Collisional activation coupled with IM-MS analysis can be used to probe changes in structure and how these couple to fragmentation for any given molecular ion.^38^,^40^–^43^ This method has been previously applied to compare the behaviour of one mAb lot as a function of glycosylation both in an intact IgG1 and an IgG1 Fc-fragment (IdeS digested below the hinge).^44^ Comparing each of the samples following sequential enzymatic truncation of the glycans (six glycosylated states in total) against the fully glycosylated mAb, Tian *et al.* demonstrated that the collisional activation energy required to trigger the conformational transitions correlated to the size of the glycan; where increased glycan size provided increased stabilization towards conformational changes.

**Fig. 3:**
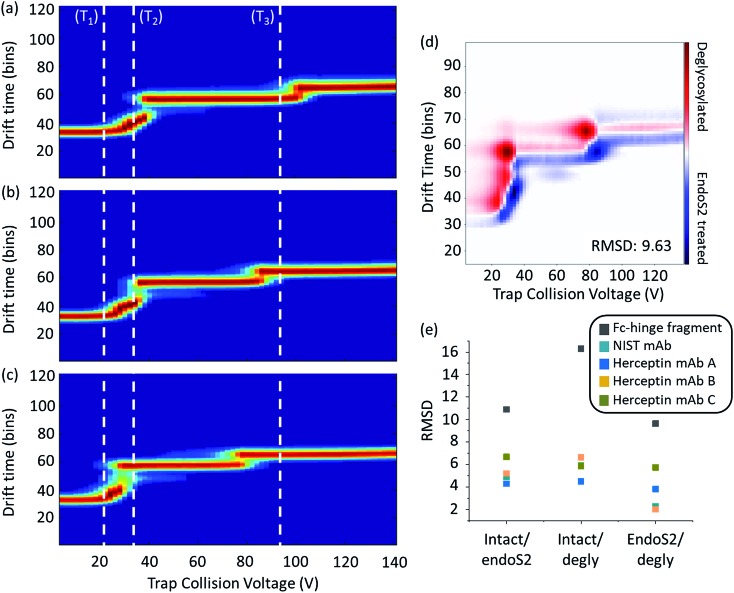
Ion mobility mass spectrometry heat maps as a function of collisional activation energy of an IgG1 Fc-hinge fragment with glycans (a), truncated (endosS2 treated) glycans (b) and no glycans (c). White dashed lines represent the start of a conformational transition (T_1_, T_2_ and T_3_) relative to the intact sample; associated quantitative collision energy values are reported in Table S2 in the ESI.† Data shown for the mass selected 14^+^ charge state with collision voltage range 4–140 V. Example activated IM-MS difference plot for the comparison of endoS2 treated and deglycosylated Fc-hinge fragments (d) along with calculated RMSD values for all samples at all levels of comparison (e). Heat maps for the IgG1 mAbs are provided in Fig. S9–S12 in the ESI.†

Here we use a similar approach to compare across Herceptin® lots as well as three levels of glycosylation. To quantify differences, ORIGAMI^38^ subtraction plots were generated along with the root-mean-square-deviation (RMSD, Fig. 3d and e). Differences in RMSD across the Herceptin® lots with the same extent of glycosylation demonstrate lot-to-lot variations (Table S1 in the ESI†). The unfolding profiles for lots A and B are highly similar evidenced by the low RMSD values. When compared with lot C however, the unfolding transitions occur at lower collision voltages indicating reduced stability towards collisional activation compared with lots A and B. The RMSD values and heat maps for the Herceptin® lots and the NIST mAb standard with varying levels of glycosylation, are summarised in Fig. 3e and ESI Fig. S9–S12.† As the extent of glycosylation is reduced the collision voltage required to induce the distinct conformational transitions is also reduced, however there is an inherent stability induced by truncation of the glycans using endoS2 *cf.* removing the glycan completely.

In order to provide more detail on the observed conformational changes we can compare the behaviour of the Herceptin® Fc-hinge fragment as a function of glycosylation and contrast it with the findings of Tian *et al.* who examined Fc/2 (Fig. 3 and Table S2 in ESI†). When fully deglycosylated the Fc-hinge fragment more readily transitions (as shown by the comparison of the inflexions to the dotted lines T_1_, T_2_ and T_3_), at collision voltages significantly lower (27–39%) than those required at the intact level. Although the stability trends are the same across the mAb samples and the Fc-hinge fragment, the increase in RMSD for the fragment sample means there is a clearer distinction between the different levels of glycosylation (Fig. 3e). We attribute these differences in RMSD magnitude to the proportion of glycosylation relative to the protein; for the intact mAb, glycosylation accounts for ∼2% mass whereas this increases to ∼6% for the IgG1 Fc-hinge fragment. Decreasing the protein size, increases the potential sensitivity of the activated IM-MS experiment and therefore enhances the relative impact of altering glycan structure. The effect of glycosylation upon Fc conformation has been well documented^17^,^45^ and with increased experimental sensitivity, structural and stabilisation effects across samples will be more apparent when compared with the intact mAb.

### What is the difference in solvent accessibility of the lots in solution and can we map it to the tertiary fold?

HDX-MS monitors the isotopic exchange of hydrogen for deuterium along the protein backbone as a mass shift with respect to analysis time.^46^ By mapping regions of solvent accessibility onto the protein structure, HDX-MS offers information regarding the tertiary fold, flexibility and average conformation of the protein in solution over an experimental timescale that can range from seconds to days.^47^ We used HDX-MS experiments to monitor the effects of varying levels of glycosylation upon the mAb dynamics of Herceptin® lots A and C, the Fc-hinge fragment and the Waters mAb as a standard. (For these experiments the NIST mAb was not used as the HDX-MS sequence coverage achieved was ∼58% compared with 70–80% achieved with the other samples). Each sample was analysed in the fully glycosylated form (intact), after endoS2 digestion and following deglycosylation. Observations made across all samples were that for most peptides (81%) deuterium uptake increased according to the trend, intact < deglycosylated < endoS2 treated (see ESI Fig. S13 and S14† for examples). For the Herceptin® mAb lots, the uptake difference between the three sample preparations was generally greater than for the Waters mAb standard; with reduced comparability between the endoS2 treated mAb peptides and the deglycosylated mAb peptides from the mAb standard (ESI Fig. S13 and S14†). The complete peptide uptake difference plots for the Waters mAb standard (ESI Fig. S15†) confirm the increased deuterium uptake in the absence of part or full glycan moieties, with minimal difference between the two.

**Fig. 4:**
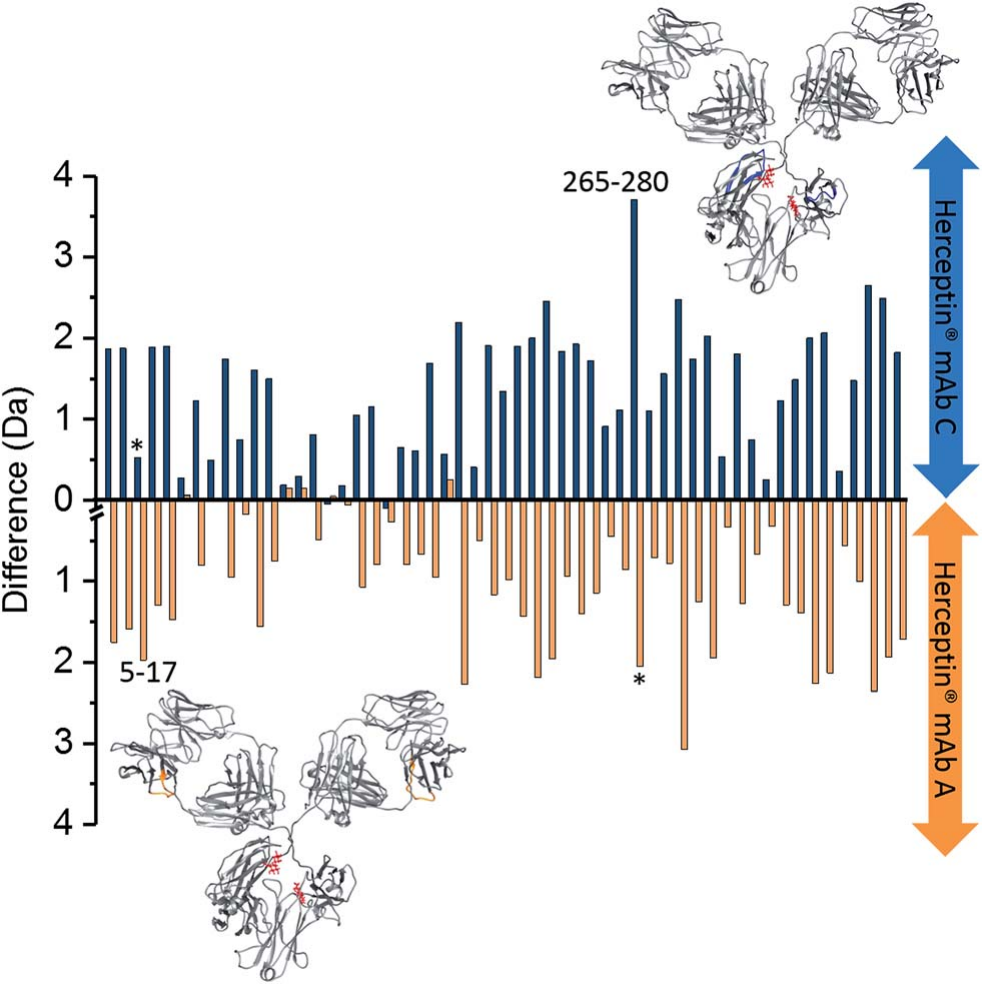
Deuterium uptake difference plots for heavy chain (HC) intact *vs.* endoS2 treated Herceptin®; endoS2–intact. Each bar represents a different peptide (HC sequence coverage = 70.2%, 55 peptides). Blue bars represent the deuterium uptake differences for Herceptin® lot C; orange bars represent deuterium uptake differences for Herceptin® lot A. Labelled peptides 5–17 (VESGGGLVQPGGS) and 265–280 (VVVDVSHEDPEVKFNW) are those with significant uptake differences between the two Herceptin® lots *i.e.* a difference of >1 Da (see ESI Fig. S16† for the Herceptin® lot C–lot A subtraction plot); * corresponds to the equivalent peptide in the other sample. The peptide locations for the two peptides with uptake differences >1 Da are highlighted on the mAb structures in blue and orange for lot C and lot A, respectively (PDB: ; 1IGY). A representation of endoS2 treated glycans are shown in red. Subtraction of intact mAb data from enzyme treated mAb data for individual lots, means that the intact data serves as a control to counteract any day-to-day variations in the HDX setup. An interactive version of this figure is available at the following link; ; http://bit.ly/2OjV3R6.

A comparison of intact *vs.* endoS2 treated Herceptin® (Fig. 4), was made by subtracting the endoS2 uptake data from the intact uptake data for each identified peptide along the heavy chain for both Herceptin® lots A and C independently. Subtracting within individual samples enables direct comparison between samples as the results are independent of differences in intact uptake *i.e.* provided a direct comparison of the effects of endoS2 treatment upon the intrinsic stability of the Herceptin® lots. In both Herceptin® lots there was a significant increase in uptake across the heavy chain following endoS2 digestion, implying significant structural alterations leading to the availability of more solvent accessible hydrogen atoms. A plausible explanation for this is that the glycan ‘stubs’ (single GlcNAc or [GlcNAc + Fucose]) interact with one another to ‘hold-open’ the Fc-domain, increasing the solvent accessibility without the steric hindrance of the intact glycan.

**Fig. 5:**
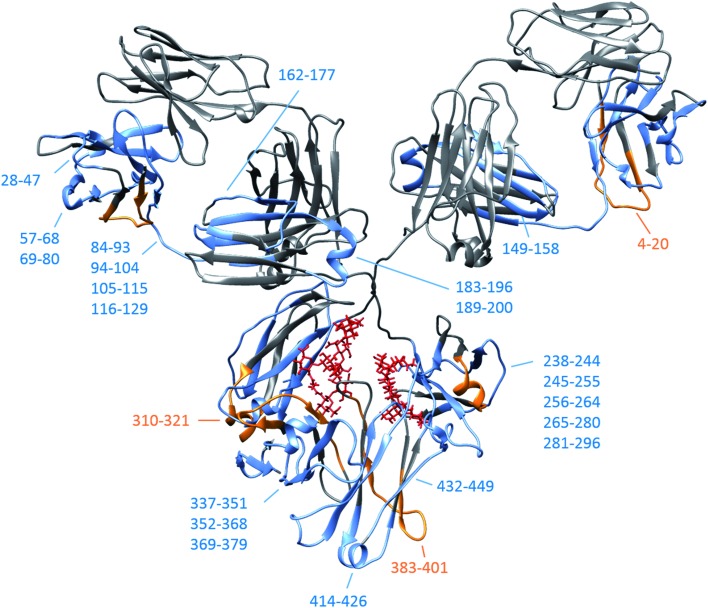
IgG1 schematic (pdb: 1IGY) depicting the location of all heavy chain peptides shown in ESI Fig. S16† for intact *vs.* endoS2 treated Herceptin® lots. Peptides/consecutive peptide regions where the average greatest uptake is in lot C are shown in blue and are shown in orange for peptides from lot A. Original intact glycan positions shown in red. Heavy chain peptide coverage maps are provided in ESI Fig. S21 and S22.†

By subtracting the two sets of data presented in Fig. 4 from one another, differences between Herceptin® lot C and lot A were seen (ESI Fig. S16†), with the majority of peptides showing an increased deuterium uptake in Herceptin® lot C compared with lot A. The peptides with most significant differences (peptides 5–17 and 265–280) are highlighted on the mAb structures in Fig. 4 (and in ESI Fig. S16†) with the location of all peptides shown in Fig. 5. Peptides close to the site of glycosylation (281–296 and 310–321 for example) were understandably affected by the truncation of the glycans, and interestingly so were a number of peptides in the Fab region; implying that alterations to the Fc region conformation also causes conformational changes in the Fab region. Intact *vs.* deglycosylated heavy chain data and endoS2 *vs.* deglycosylated heavy chain data are shown in ESI Fig. S17 and S18,† respectively. There are clear similarities between ESI Fig. S16 and S17,† confirming that both glycan truncation and removal increases deuterium uptake to a greater extent in Herceptin® lot C compared with lot A. The quantitative differences between the two glycosylation levels are presented in ESI Fig. S18; † no significant differences are observed (at the >1 Da level), however nearly all peptides in Herceptin® lot C show increased uptake compared with lot A.

These same uptake trends were observed in the Herceptin® light chain data and for the Fc-hinge fragment (ESI Fig. S19 and S20,† respectively). The Fc-hinge fragment, yields similar HDX data from both endoS2 treated and deglycosylated samples, with four of the five peptides/regions with significant uptake differences (>1 Da) in both treated samples compared with the intact sample. For the endoS2 treated Fc-hinge fragment an additional peptide (166–183) was significant (ESI Fig. S20†). All HDX-MS data was analysed in duplicate with the same uptake trends observed across all repeats and samples.

### Orthogonal techniques to assess lot-to-lot variability

Disulphide bond mapping was performed on each of the Herceptin lots to confirm that the conformational differences discussed above were not as a result of non-canonical cysteine pairings. A comparison of the proteolytic digests from the alkylated and non-alkylated samples identified the likely disulphide bonds within the starting material (Table S3 in the ESI†). The Lys-C digest revealed no differences in the protein sequences, no deviations from the canonical disulphide bridge pattern expected and no PTM variations across the lots.

Circular dichroism was used to assess the secondary structure of the Herceptin lots and to allow comparison with the NIST mAb. Following conversion to molar ellipticity units, unlike the MS approaches described above this method did not reveal any observable differences between the lots (Fig. S23 in the ESI†).

## Conclusions and outlook

We have shown that native mass spectrometry can be usefully employed to distinguish between lots of an active biopharmaceutical and demonstrated a method by which data can be compared with that obtained from a standard. The results present significant conformational similarities between two of the Herceptin® lots however, lot C exists in a wider range of conformational states demonstrated by both HDX-MS and IM-MS. We have also mapped how variations in N-linked Fc-domain glycosylation affect the intrinsic conformations of IgG1 mAbs. HDX-MS shows how deglycosylation causes more solvent exposure in both the Fc and Fab domains, and *in vacuo* analysis indicates a concomitant conformational contraction/collapse and higher flexibility upon collisional activation of the mAb. Similar observations are made for endoS2 treated samples, compared with the intact glycosylated samples, although the deuterium uptake for the majority of peptides was greater than for the equivalent deglycosylated samples. Taken together these findings indicate that the truncated glycan ‘stubs’ (single GlcNAc or [GlcNAc + Fucose]) interact within the Fc-domain, creating a solvent accessible pocket with reduced steric hindrance. The IM-MS data supports this also with the widest conformational spread being observed for the endoS2 treated Fc-hinge fragment and with the retention of some stability demonstrated by the activated IM-MS heat maps. As for the comparisons across different lots of Herceptin®, the conformational trends (endoS2 treated > deglycosylated > glycosylated) across the techniques are the same. However the magnitudes differ particularly for Herceptin® lot C. With all three lots having approval for clinical use from the relevant authorities, these findings indicate the range of measurable conformational properties that may fulfil acceptance criteria. With this in mind it is no trivial matter defining all critical quality attributes for complex biopharmaceuticals, and full characterisation of originator lots should be coupled with clinical consequences, for example the level of afucosylation, before embarking upon biosimilar classification. In this work we have considered three lots of Herceptin® and three levels of glycosylation (intact, truncated and fully deglycosylated) and going forward it would be desirable to extend this approaches to explore a wider range of glycan occupancies. Such studies might coupled sequential enzymatic cleavages to the glycan structure of the mAb with *in vitro* cell-based bioassay and surface plasmon resonance (SPR) techniques to assess the effects upon therapeutic activity as we have previously demonstrated in comparing originator lots with research grade material.^26^ In addition it would be interesting to evaluate how these techniques complement and/or support data from orthogonal techniques. For example, the IM-MS CIU transitions could be correlated with thermal stability data obtained by DSC. We recommend that native MS methods could have a very useful role for initial characterisation as they are rapid and lend well to comparability studies. Currently the complexity of the HDX-MS data generated and the significant cost of both machine and analysis time means that this technique is not yet amenable to high-throughput activities such as discovery phase screening for complex biotherapeutics. However, as the data processing packages and technology improve, the techniques will not only become complementary to established DSC and SEC methods for example, but may have the ability to supersede these techniques in the future. Statistical significance analysis is an avenue already being explored which will inevitably aid interpretation of HDX-MS data and help increase the specificity of the technique.^48^,^49^ Analysis of biosimilar candidates in comparison with a wider range of originators would also help cement these techniques and the information provided within a robust, regulatory biosimilar characterisation workflow.

## Conflicts of interest

There are no conflicts to declare.

## Supplementary Material

Supplementary information
