# Supplementary material for: Hybrid mass spectrometry methods reveal lot-to-lot differences and delineate the effects of glycosylation on the tertiary structure of Herceptin®
Source: Chem Sci. 2019 Jan 17;10(9):2811–20. doi: 10.1039/c8sc05029e (PMC6425993; doi:10.1039/c8sc05029e)
Supplement: Supplementary file 1 [file SC-010-C8SC05029E-s001.pdf]

Electronic Supplementary Material (ESI) for Chemical Science.
This journal is © The Royal Society of Chemistry 2019

Supporting Information

Hybrid Mass Spectrometry Methods Reveal Lot-to-Lot Differences and Delineate the Effects of Glycosylation on the Tertiary Structure of Herceptin

Rosie Upton[1,2], Lukasz G. Migas[1], Kamila J. Pacholarz[1], Shirley J. Peters[3], Rachel A. Davis,[3] Richard G. Beniston[2], Sian Estdale[2], David Firth[2]and Perdita E. Barran[1]

[1]Manchester Institute of Biotechnology, Michael Barber Centre for Collaborative Mass Spectrometry, University of Manchester, 131 Princess Street, Manchester, M1 7DN, United Kingdom

[2]Covance Laboratories Ltd., Otley Road, Harrogate, HG3 1PY, United Kingdom

[3]UCB Pharma, 216 Bath Road, Slough, SL13WE, United Kingdom

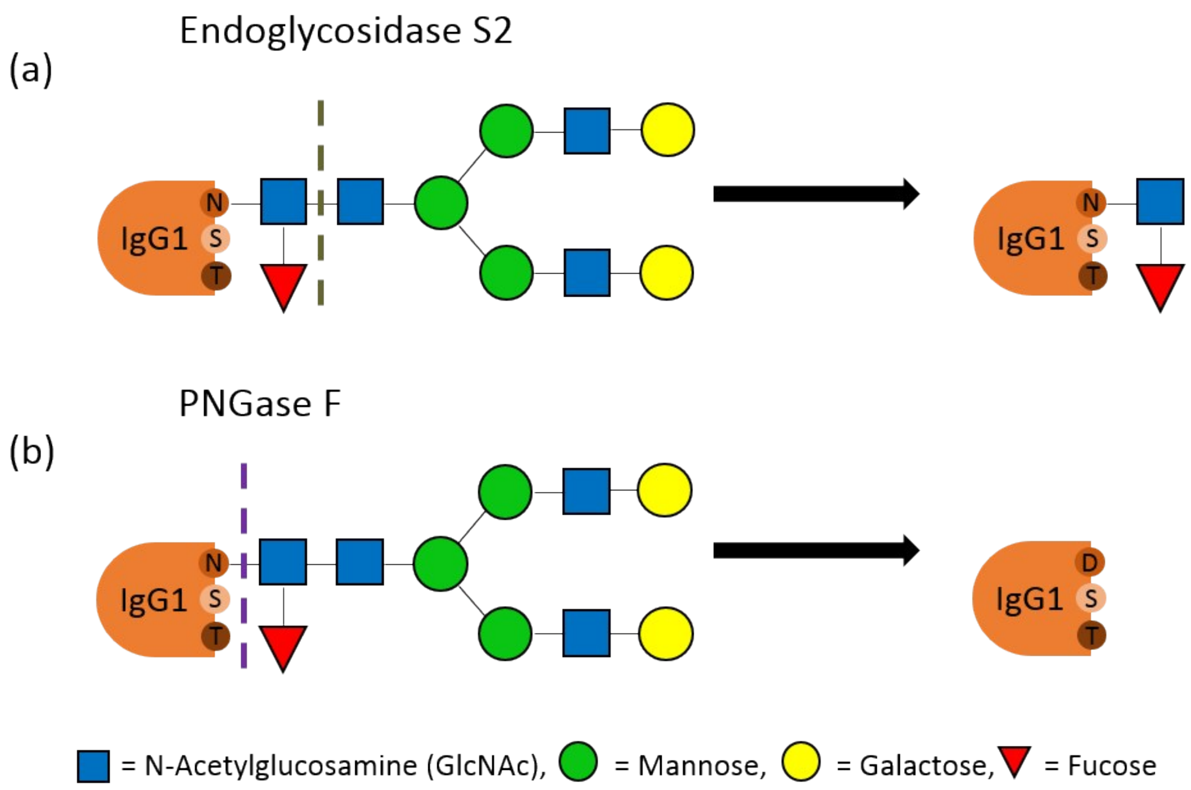

Figure S1: Enzymatic cleavage of IgG1 glycans; (a) endoglycosidase S2 cleaves between the two GlcNAc residues in the chitobiose core and (b) PNGase F cleaves off the entire N-linked glycan, converting asparagine (N) to aspartic acid (D) which results in a +1 Da mass shift. Glycan G2F used as an example. Glycan not to scale.

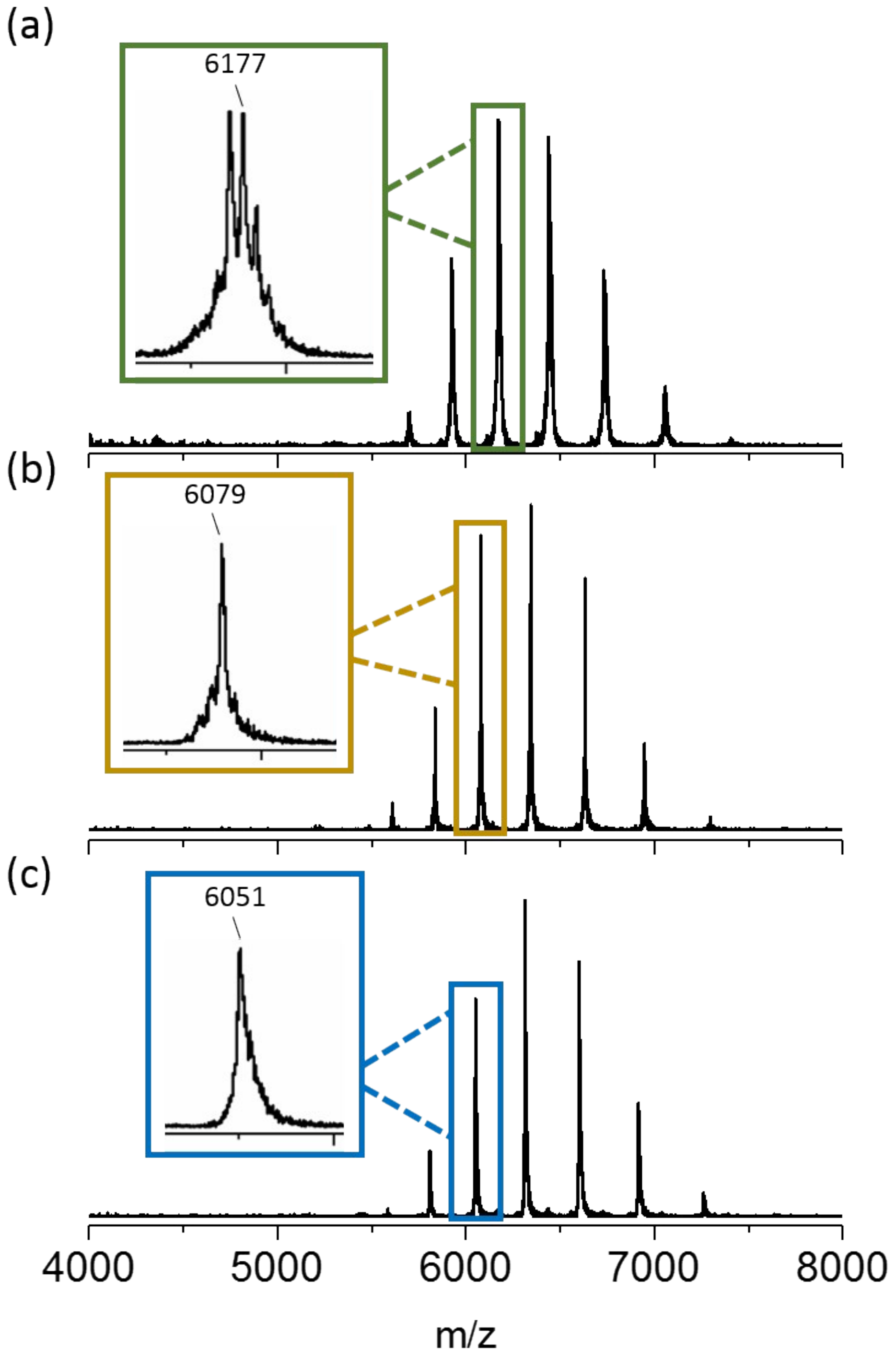

Figure S2: Mass spectra for Herceptin® (a) fully glycosylated (intact), (b) following endoS2 treatment (truncated glycans) and (c) following PNGase F treatment (fully deglycosylated). The inserts are a zoom-in of the 24⁺ charge state. This Figure is available as an interactive Figure at the following link: http://bit.ly/2OicQIi.

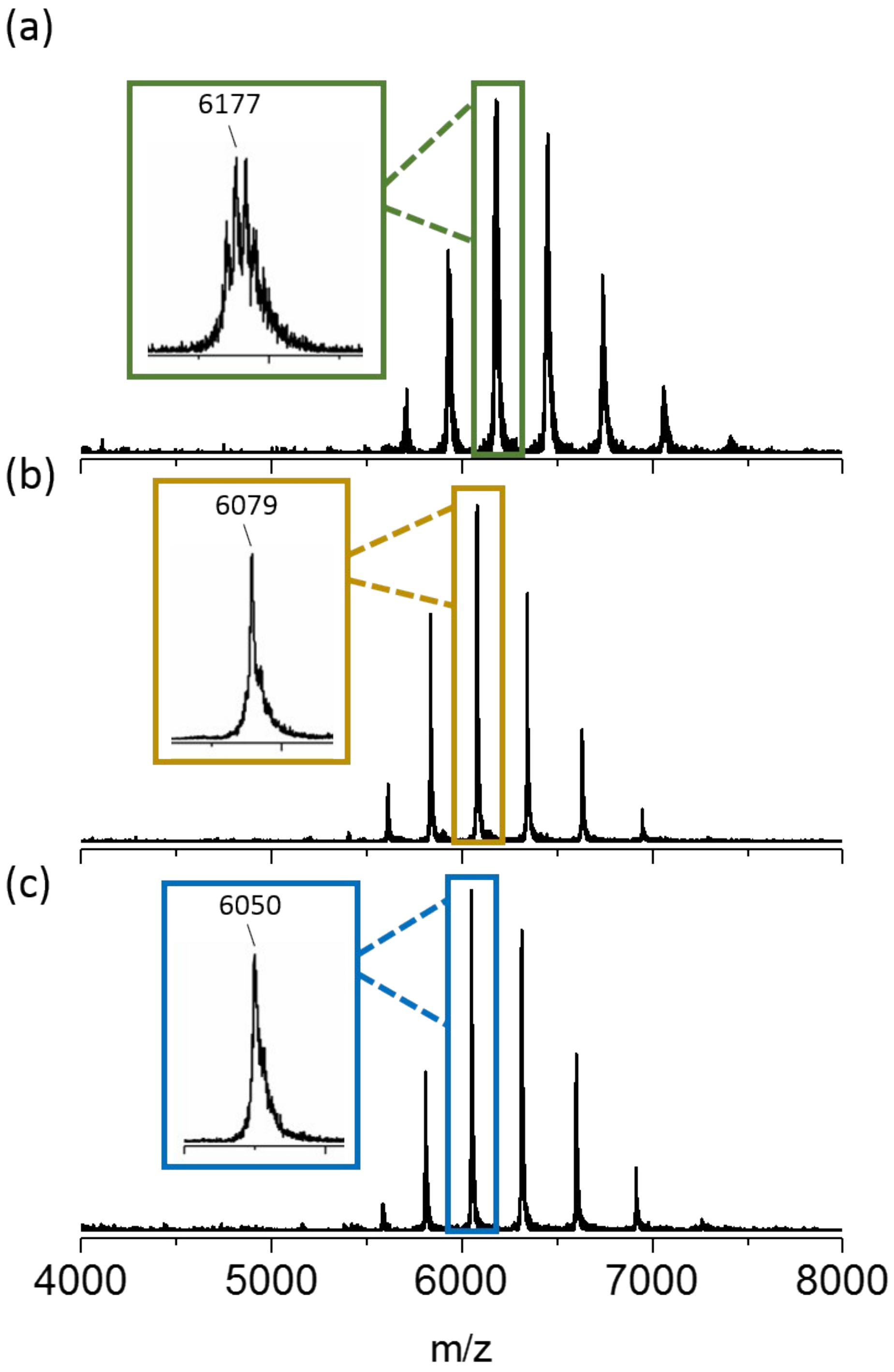

Figure S3: Mass spectra for the NIST mAb standard (a) fully glycosylated (intact), (b) following endoS2 treatment (truncated glycans) and (c) following PNGase F treatment (fully deglycosylated). The inserts are a zoom-in of the 24⁺ charge state. This Figure is available as an interactive Figure at the following link: http://bit.ly/2LVjanl.

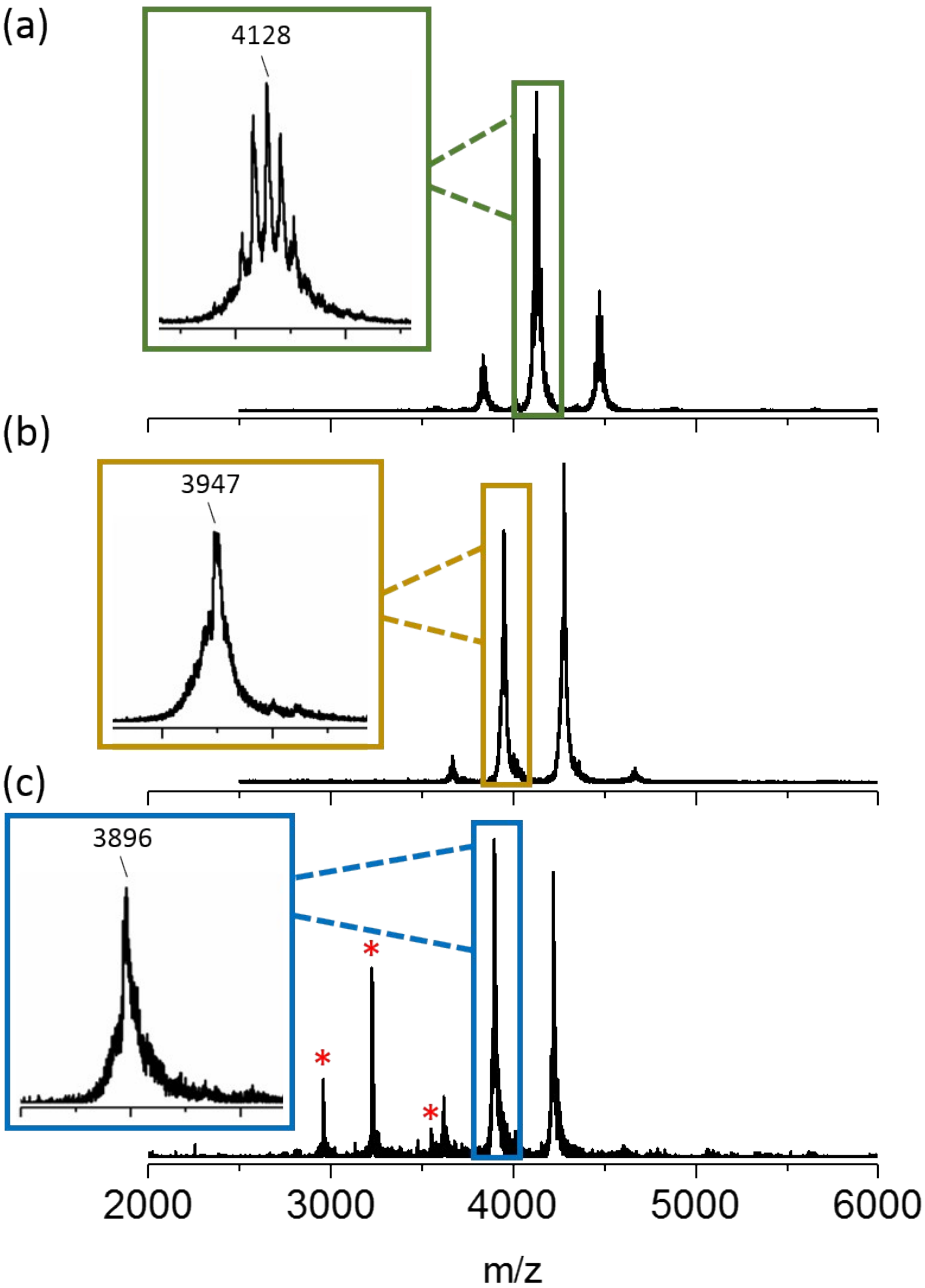

Figure S4: Mass spectra for the IgG1 Fc-hinge fragment (a) fully glycosylated (intact), (b) following endoS2 treatment (truncated glycans) and (c) following PNGase F treatment (fully deglycosylated). The inserts are a zoom-in of the 13^{+} charge state. This Figure is available as an interactive Figure at the following link: http://bit.ly/2NOgSHq. The red asterisk denotes PNGase F peaks.

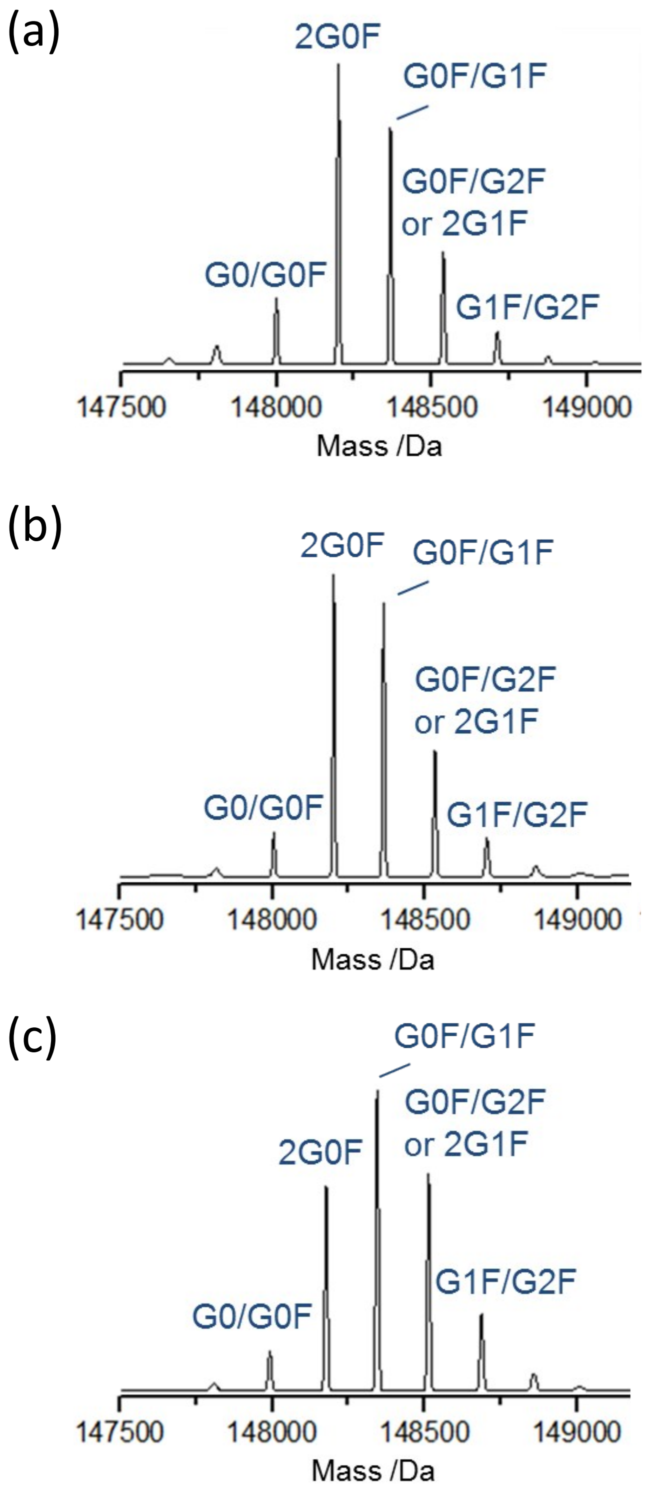

Figure S5: Deconvoluted mass spectra associated with (a) Herceptin lot A, (b) Herceptin lot B and (c) Herceptin lot C. Peak assignments based upon comparison of MaxEnt1 deconvolution data with calculated values.

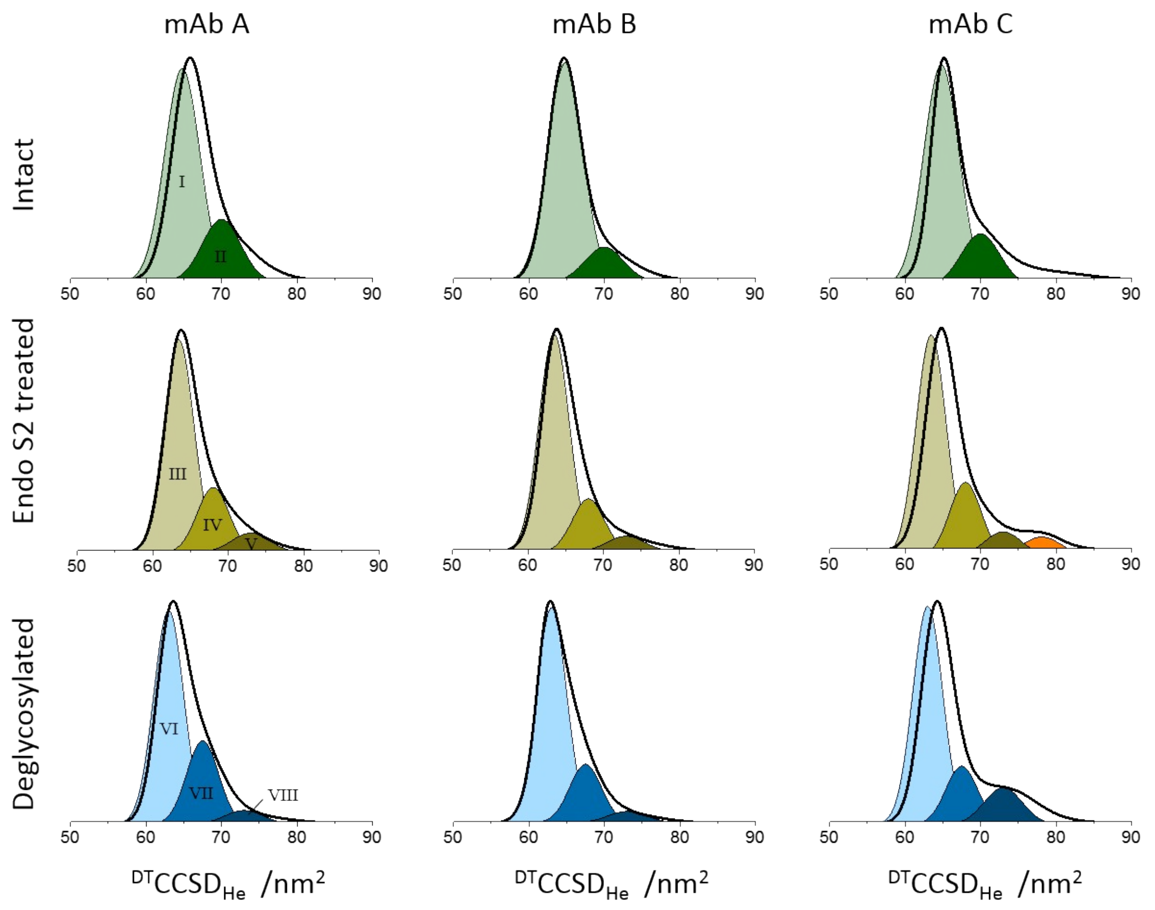

Figure S6: $^{DT}CCSD_{He}$ plots fitted with the conformers (I – VIII) defined for the IgG1 NIST mAb. Individual conformers defined for each variation of sample i.e. intact, endo S2 treated and deglycosylated. More extended conformers illustrated with darker colours. An additional conformer (most extended) only present in the endo S2 treated Herceptin® lot C sample is shown in orange.

The Herceptin® mAb lots (A, B and C) and NIST mAb reference standard were deglycosylated using PNGase F and digested separately using endo S2 then analysed via linear IM-MS in helium. The

measured arrival time distributions (ATDs) were converted to $^{DT}CCS_{He}$ distributions (CCSDs) for the most intense charge states (7). The change in CCS between intact and glycan treated samples was greater (~3.2-3.5%) than the corresponding reduction in mass of ~2%, therefore confirming that glycan removal alters conformation. For all samples there was a decrease in both the width and position of the CCSD plots for charge states 22^+ - 24^+ with endo S2 digestion and PNGase F treatment. The 25^+ charge state in particular offered clear differences between the samples, with distinct second conformers and shifts to higher CCS. Samples A and B showed a high degree of similarity across all charge states, both in terms of the charge state distributions presented and the changes in conformation induced upon glycan removal. The NIST standard and sample C, however, presented quite different conformational pictures. In both samples there was clearly a more prominent extended distribution for the 25^+ charge state indicative of a more flexible conformational spread; confirmed also by the increase in standard deviation.

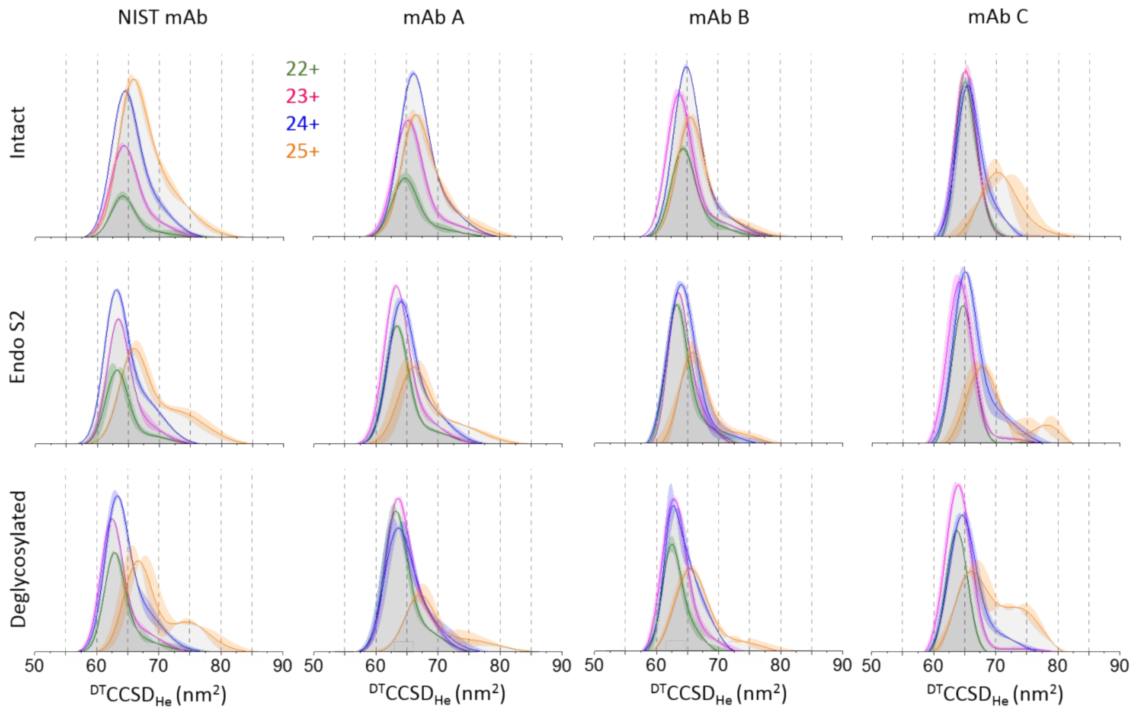

Figure S7: $^{DT}CCSD_{He}$ plots for fully glycosylated (intact), partially deglycosylated (endo S2 treated) and fully deglycosylated (PNGase F treated) IgG1 mAbs (charge state range 22^+ to 25^+). Data fitted with Gaussians (cumulative fit plotted) and normalised against (IMS area fraction) x (MS peak height fraction). Width of shading around each peak denotes the standard deviation calculated, n=3. An interactive version of this Figure is available through the following link <u>http://bit.ly/2uQ7eNi</u>.

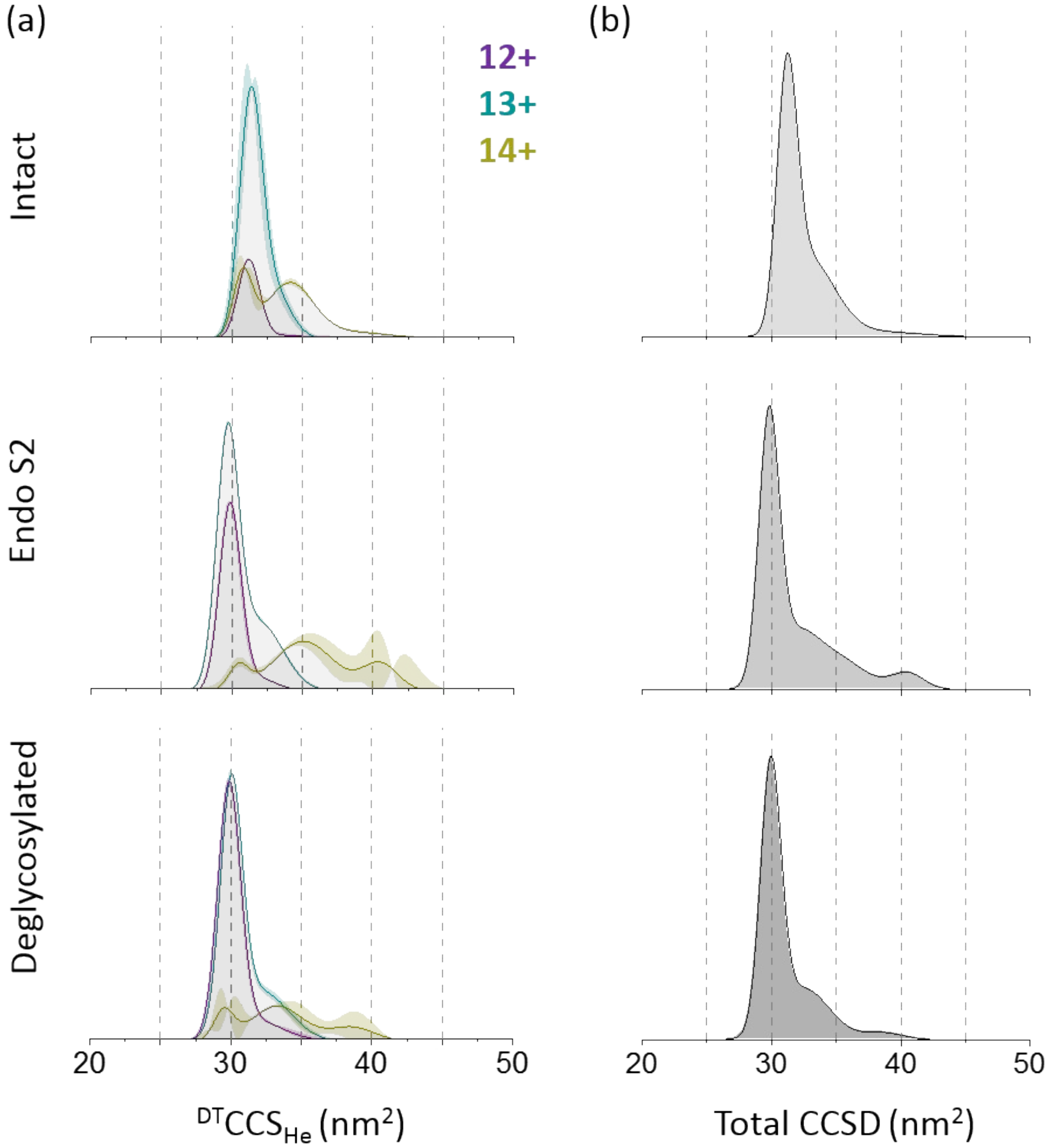

Figure S8: (a) $^{DT}CCSD_{He}$ plots for an IgG1 Fc-hinge fragment with full glycans (intact), truncated glycans (endo S2 treated) and no glycans (deglycosylated); charge state range 12^+ to 14^+. Data fitted with Gaussians (cumulative fit plotted) and normalised against (IMS area fraction) x (MS peak height fraction). Width of shading around each peak denotes the standard deviation calculated between three replicates. (b) Total CCSD plot for each Fc-hinge fragment preparation.

Table S1: RMSD values to compare the batch-to-batch variability across the Herceptin® lots at the three distinct levels of glycosylation; intact, endoS2 treated and deglycosylated.

Herceptin® Lots Compared	Relative Mean Standard Deviation		
	Intact	EndoS2	Degly
Lot A/ Lot B	4.02	2.38	2.28
Lot A/ Lot C	11.29	12.32	11.83
Lot B/ Lot C	11.91	11.7	11.96

Table S2: Summary of collision voltages required to initiate the conformational transitions for the different IgG1 Fc-hinge fragment preparations.

Sample	Collision Voltage / V		
	Transition 1	Transition 2	Transition 3
Intact	23	35	94
EndoS2 treated	19	31	77
Deglycosylated	14	23	68

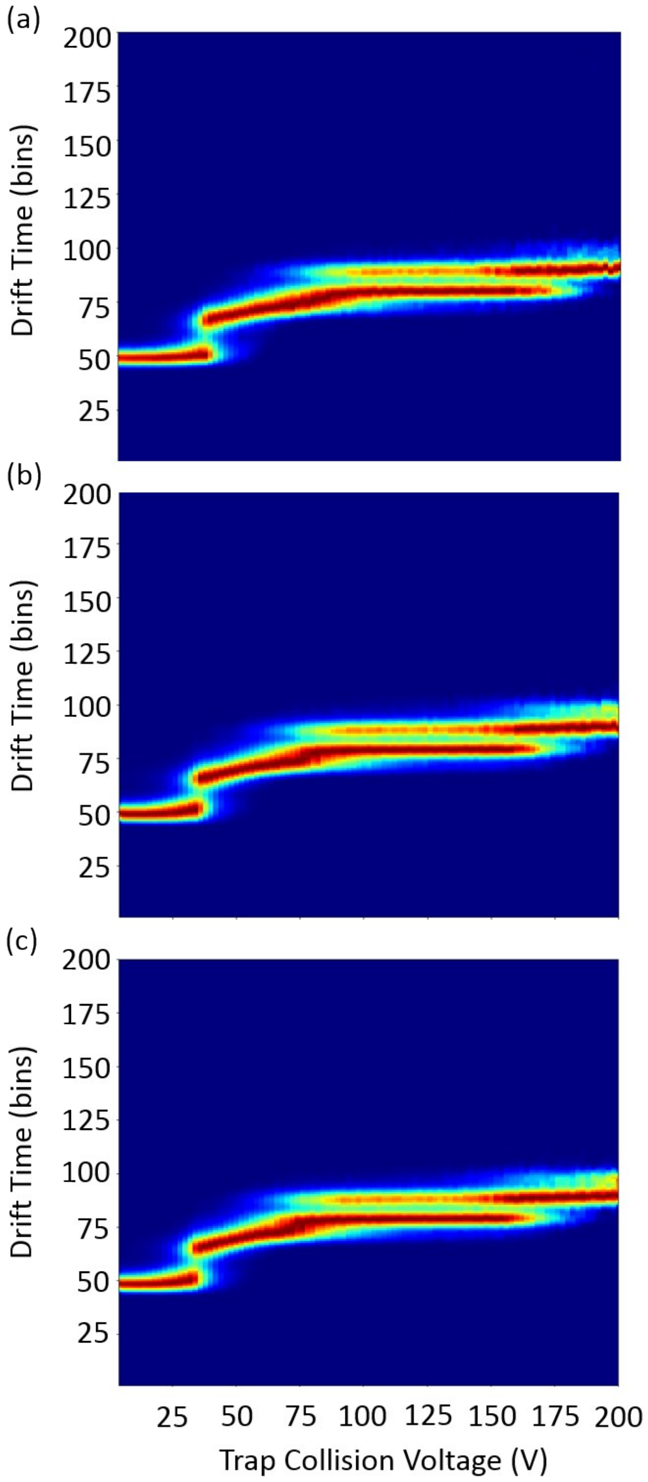

Figure S9: Collision energy activated IM-MS heat map of the IgG1 NIST mAb with (a) glycans, (b) truncated (endoS2 treated) glycans and (c) no glycans. Mass selected 24^{+} charge state with collision voltage range 4-200 V, n=3. An interactive version of this Figure is available via the following link: http://bit.ly/2NLoU3G.

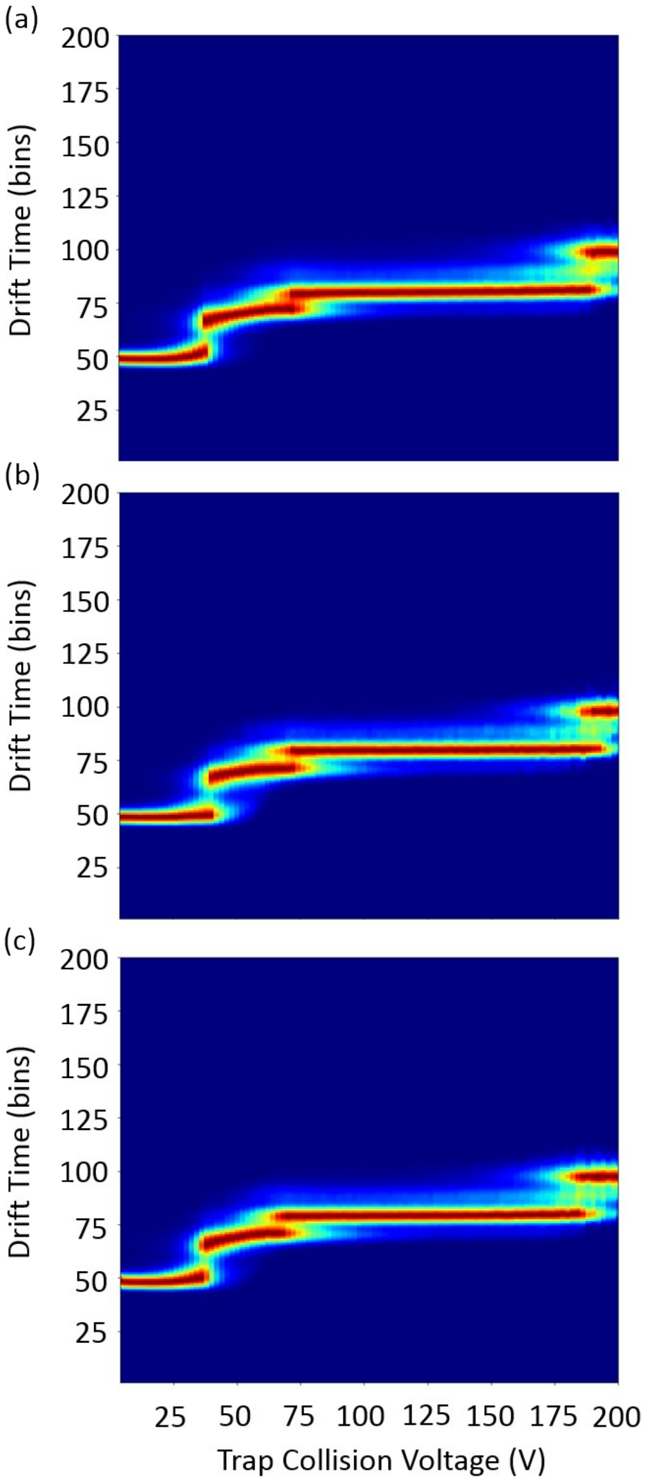

Figure S10: Collision energy activated IM-MS heat map of Herceptin® mAb lot A with (a) glycans, (b) truncated (endoS2 treated) glycans and (c) no glycans. Mass selected 24⁺ charge state with collision voltage range 4-200 V, n=3. An interactive version of this Figure is available via the following link: http://bit.ly/2NLoU3G.

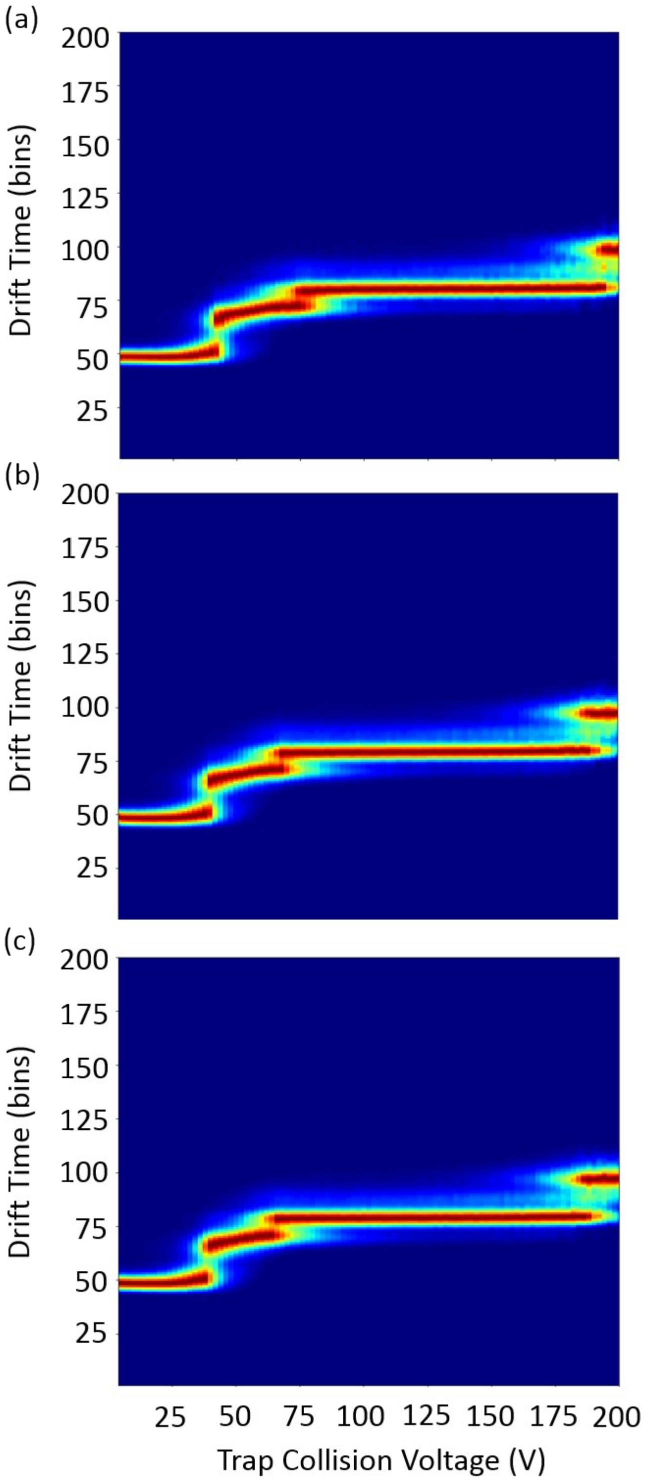

Figure S11: Collision energy activated IM-MS heat map of Herceptin® mAb lot B with (a) glycans, (b) truncated (endoS2 treated) glycans and (c) no glycans. Mass selected 24⁺ charge state with collision voltage range 4-200 V, n=3. An interactive version of this Figure is available via the following link: http://bit.ly/2NLoU3G.

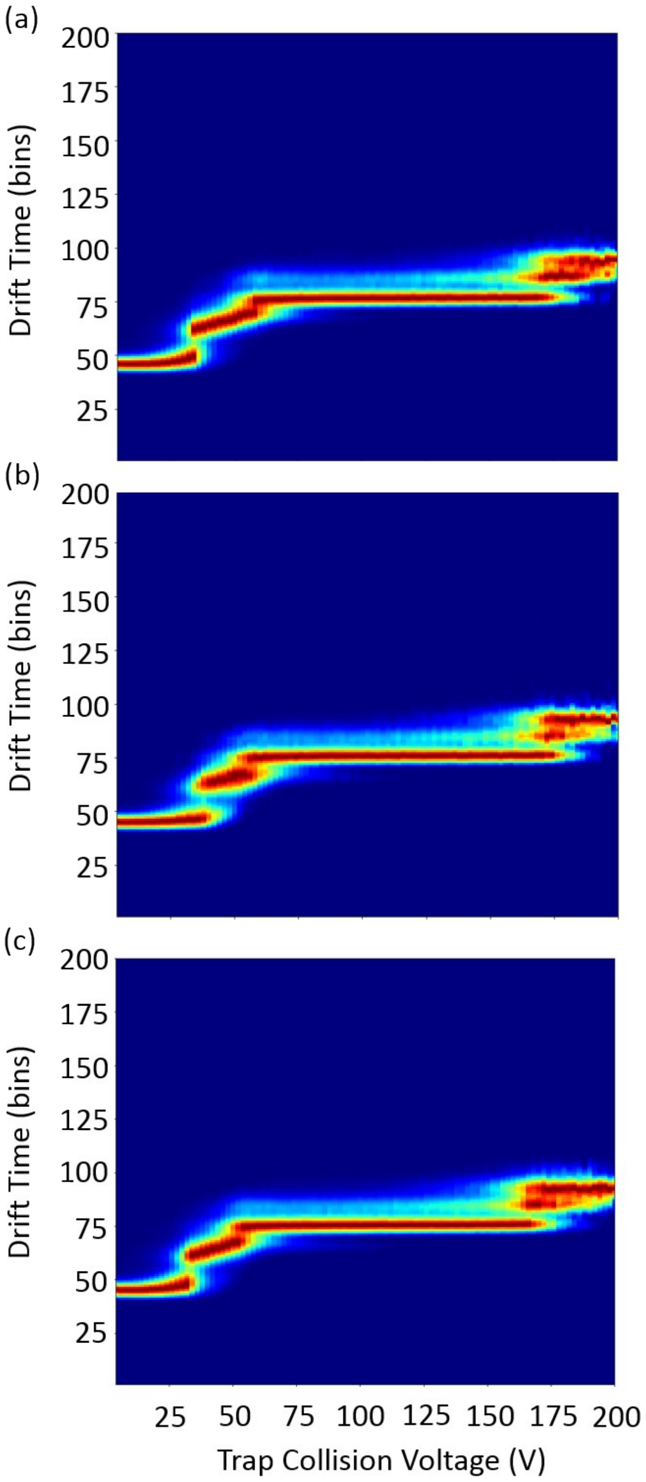

Figure S12: Collision energy activated IM-MS heat map of Herceptin® lot C with (a) glycans, (b) truncated glycans and (c) no glycans. Mass selected 24⁺ charge state with collision voltage range 4-200 V, n=3. An interactive version of this Figure is available via the following link: http://bit.ly/2NLoU3G.

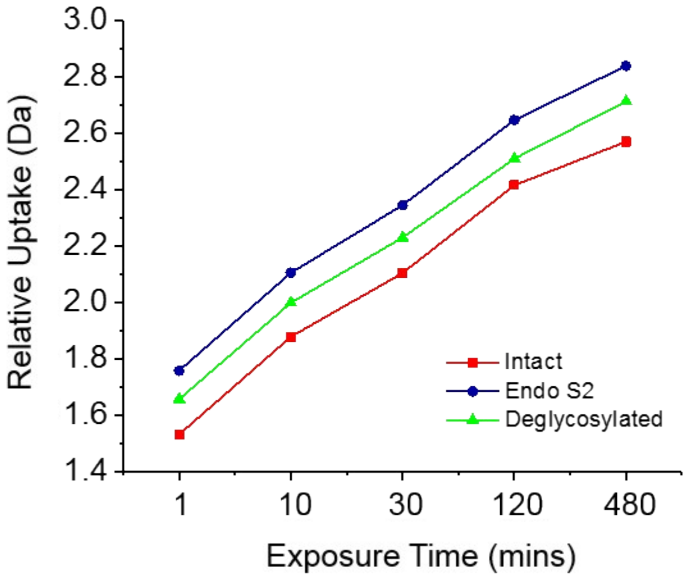

Figure S13: Typical uptake plot for an example Herceptin® mAb peptide (WESNGQPENN) highlighting the uptake differences between the three sample preparations; fully glycosylated – intact (red), partially deglycosylated – endo S2 treated (blue) and fully deglycosylated – PNGase F treated (green).

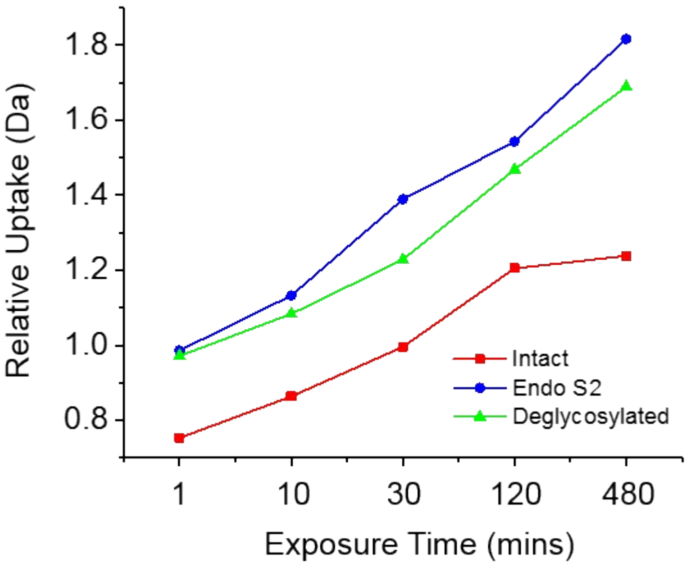

Figure S14: Typical uptake plot for an example Waters mAb peptide (HEGLHNHHTEKSLSHSPG) highlighting the uptake differences between the three sample preparations; fully glycosylated – intact (red), partially deglycosylated – endo S2 treated (blue) and fully deglycosylated – PNGase F treated (green).

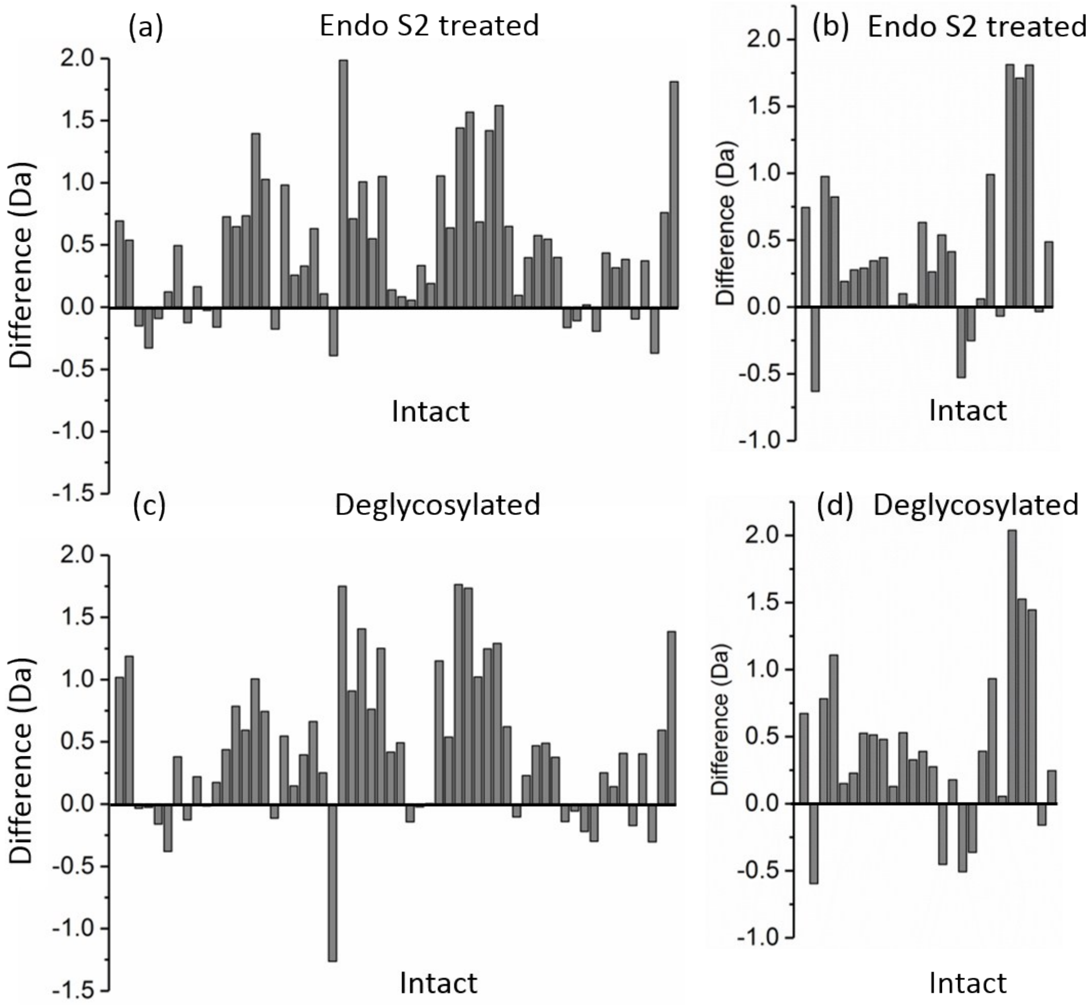

Figure S15: Deuterium difference uptake plots for the Waters mAb standard; intact vs. endo S2 treated (a) heavy chain and (b) light chain and intact vs. deglycosylated (c) heavy chain and (d) light chain. Summed uptake across 5 time points; 1 min – 8 hrs. HC coverage = 71.0% across 58 peptides, LC coverage = 82.6% across 26 peptides.

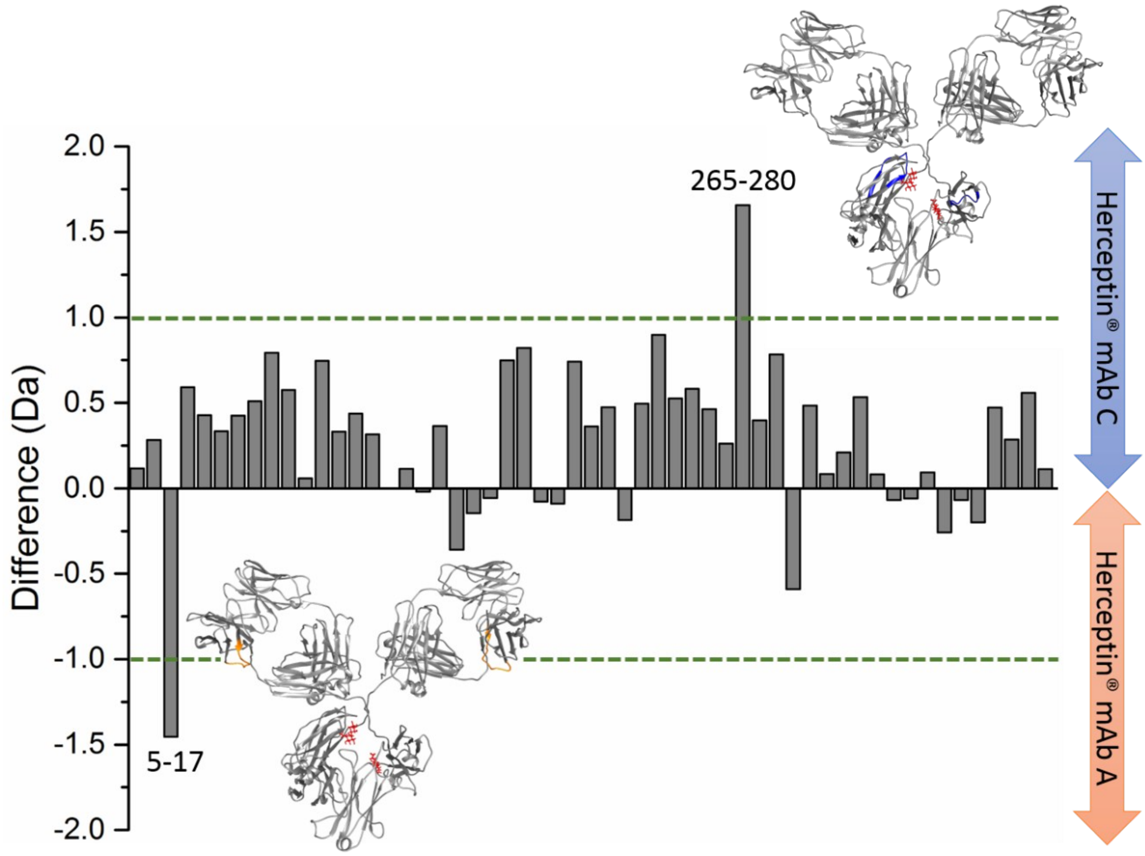

Figure S16: Deuterium uptake difference plot to compare Herceptin® lot A and lot C at the heavy chain intact vs. endoS2 treated level; lot C - lot A (subtraction of orange bars from blue bars in Figure 5 in the main text). Individual peptides exceeding the significance level of 1 Da (dashed green line) are labelled (5-17 and 265-280) and highlighted on the mAb structures in blue and orange for lot C and lot A, respectively. Summed uptake across 5 time points; 1 min – 8 hrs. HC coverage = 70.2% across 55 peptides.

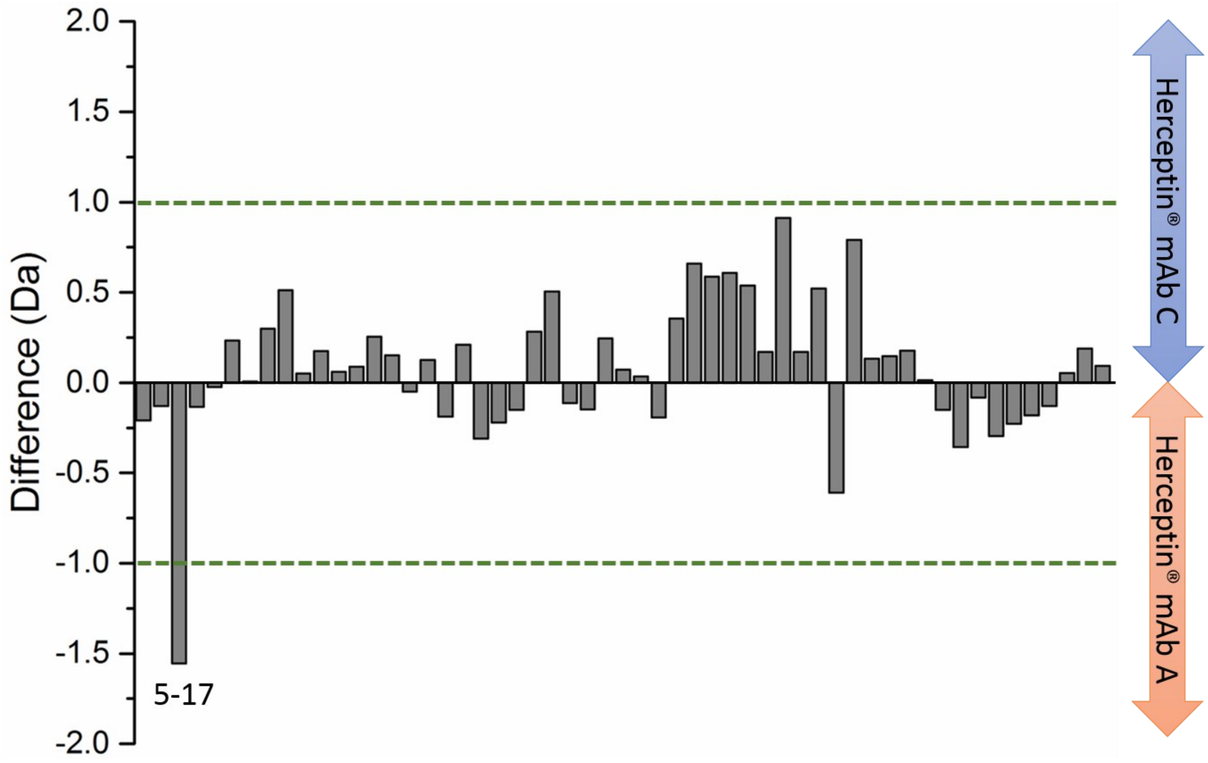

Figure S17: Deuterium uptake difference plot for heavy chain intact vs. deglycosylated; Herceptin® lot C - Herceptin® lot A. Green dashed line denotes the level of significance i.e. ± 1 Da. Peptides with greater uptake in Herceptin® lot C are positive and Herceptin® lot A peptides with greater uptake are negative. Summed uptake across 5 time points; 1 min – 8 hrs. HC coverage = 70.2% across 55 peptides.

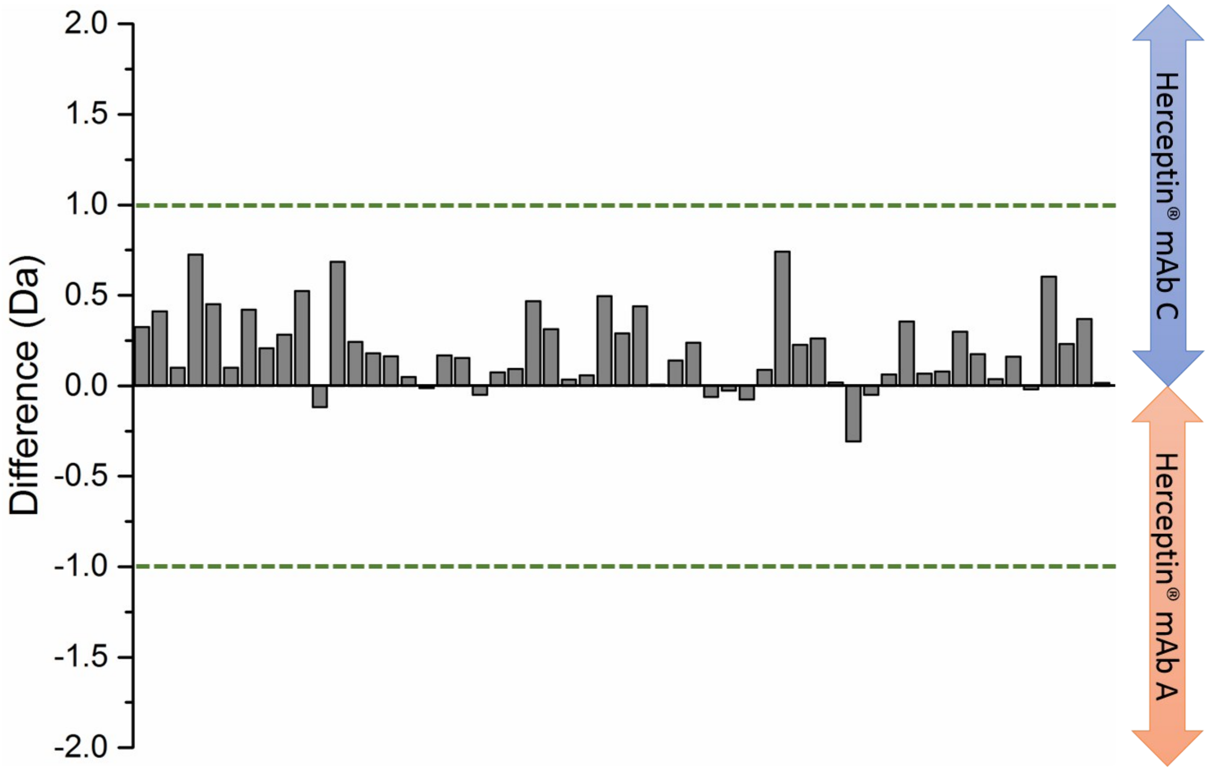

Figure S18: Subtracted deuterium uptake plot for heavy chain endo S2 treated vs. deglycosylated; Herceptin® lot C - Herceptin® lot A. Green dashed line denotes the level of significance i.e. ± 1 Da. Peptides with greater uptake in the Herceptin® lot C sample are positive and Herceptin® lot A peptides with greater uptake are negative. Summed uptake across 5 time points; 1 min – 8 hrs. HC coverage = 70.2% across 55 peptides.

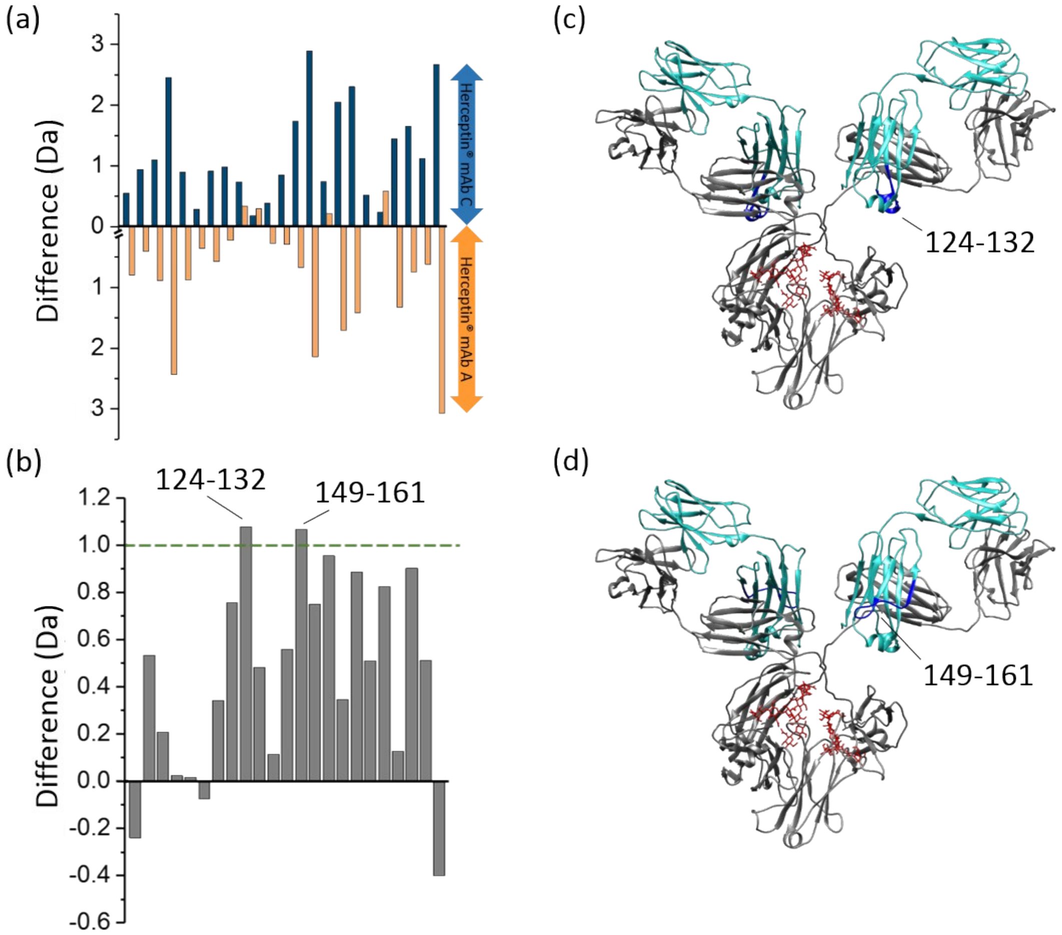

Figure S19: (a) Subtracted deuterium uptake plots for light chain intact vs. endo S2 treated Herceptin® lot A and lot C; endo S2 - intact. Each bar represents the summed uptake across 5 time points (1min – 8hrs) for a single peptide. Blue bars represent the deuterium uptake differences between intact and endoS2 treated peptides for Herceptin® lot C; orange bars represent Herceptin® lot A. (b) Blue bars minus orange bars (Figure S19a) to give a comparison between Herceptin® mAbs; lot C – lot A, at the intact vs. endoS2 level. The labelled peptides exceed the significance threshold of > 1 Da (green dashed line). The locations for the two peptides with uptake differences > 1 Da are highlighted on the mAb structures in blue; (c) 124-132 and (d) 149-161. LC sequence coverage = 67.8%, 23 peptides. Cyan denotes light chain, grey denotes heavy chain.

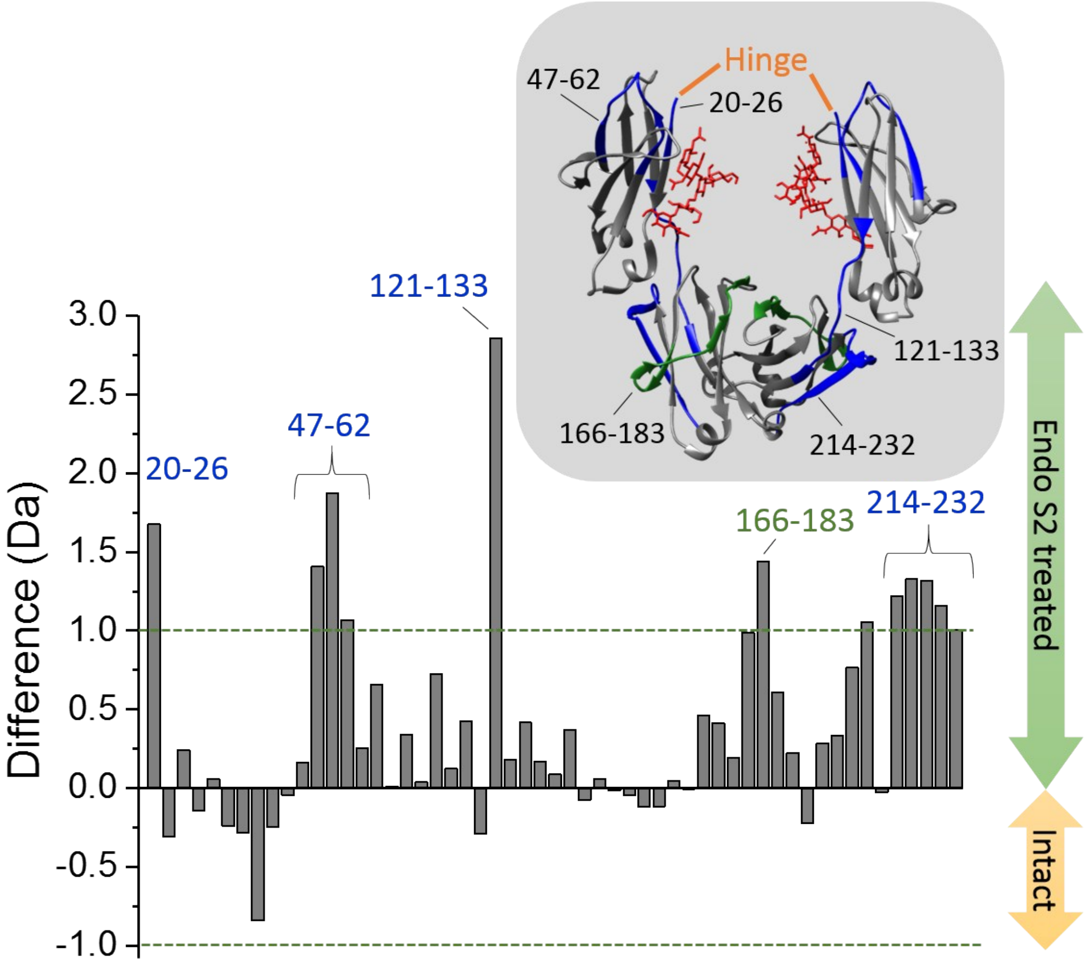

Figure S20: Difference uptake plot for the IgG1 Fc-hinge fragment endoS2 treated vs. intact. Green dashed line denotes the significant uptake threshold of 1 Da. The peptides with significant uptake in both the deglycosylated and endoS2 samples compared with the intact sample are highlighted on the Fc structure (pdb: 5JII) in blue. The peptide annotated in green only exceeded the significance threshold in the endoS2 sample. Hinge represented in orange.

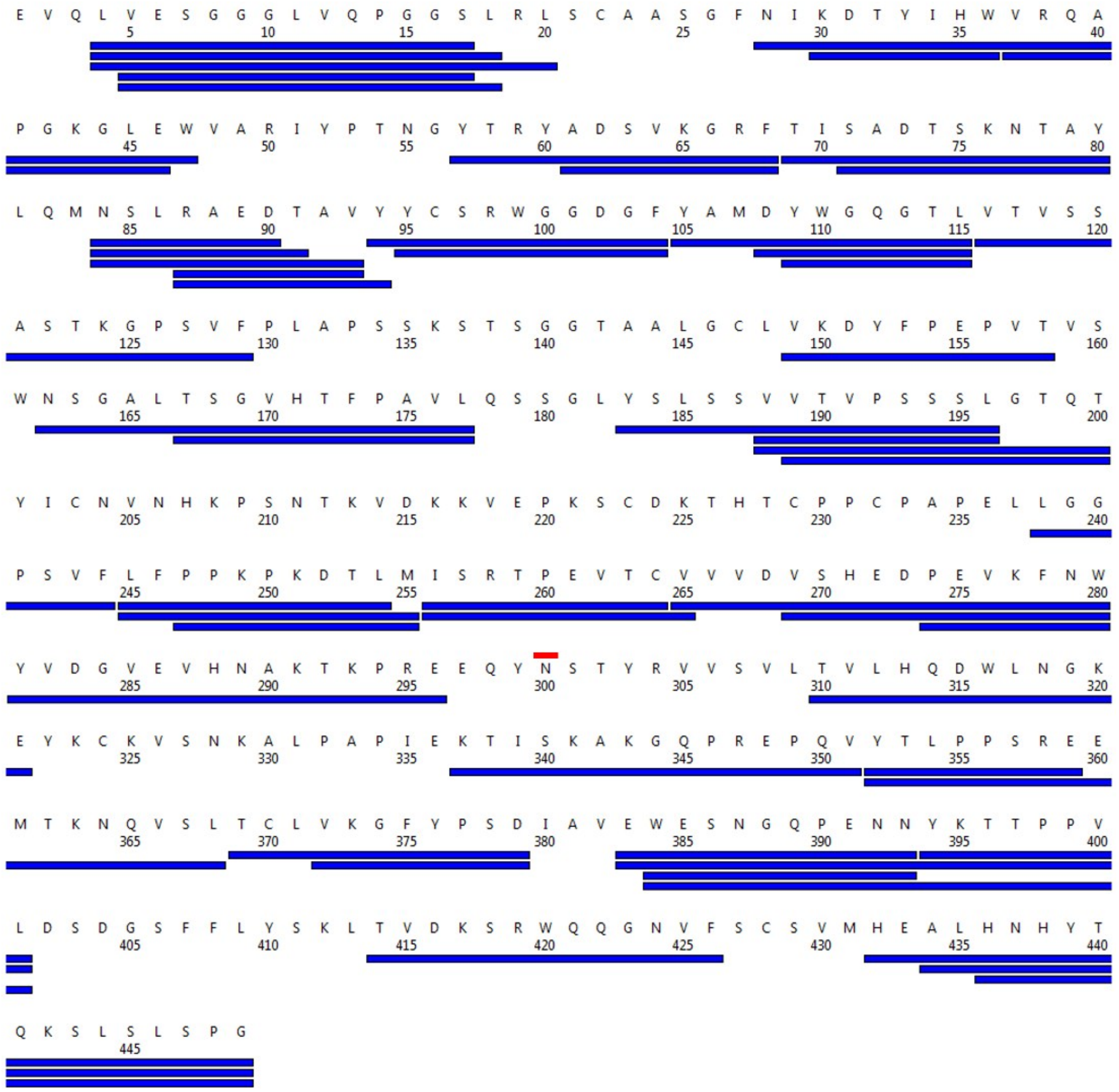

Figure S21: Peptide coverage map of glycosylated Herceptin® mAb heavy chain at t=0 (DynamX, Waters, UK). The peptides common to both Herceptin® lots A and C and across all levels of glycosylation are shown. Site of N-linked glycosylation is highlighted with a red bar (Asn-300). Total coverage = 70.2 % across 55 peptides.

4 20 28 47 57

EVQLVESGGGLVQPGGSLRLSCAASGFNIKDTYIHWVRQAPGKGLEWVARIYPTNGYTRYA

80 84

DSVKGRFTISADTSKNTAYLQMNSLRAEDTAVYYCSRWGGDGFYAMDYWGQGTLVTVSSA

129 149 158 162 177 183

STKGPSVFPLAPSSKSTSGGTAALGCLVKDYFPEPVTVSWNSGALTSGVHTFPAVLQSSGLYSL

200 238

SSVVTVPSSSLGTQTYICNVNHKPSNTKVDKKVEPKSCDKTHTCPPCPAPELLGGPSVFLFPP

hinge 296

KPKDTLMISRTPEVTCVVVDVSHEDPEVKFNWYVDGVEVHNAKTKPREEQYNSTYRVVSVL

310 321 337

TVLHQDWLNGKEYKCKVSNKALPAPIEKTISKAKGQPREPQVYTLPPSREEMTKNQVSLTCLV

379 383 401 414 426 432

KGFYPSDIAVEWESNGQPENNYKTTPPVLDSDGSFFLYSKLTVDKSRWQQGNVFSCSVMHE

449

ALHNHYTQKSLSLSPG

Figure S22: Heavy chain sequence of Herceptin® with the peptides/ consecutive peptide regions identified on Figure 4 (main text) highlighted. Peptides where the average uptake is greatest in lot C are shown in blue and are shown in orange for peptides from lot A. Site of N-linked glycosylation is highlighted with a red bar and the hinge region is shown in green.

*Table S3: Summary of the disulphide bonds identified in Herceptin® Lots A, B and C following endoS2 treatment, PNGase F treatment (deglycosylated) and no treatment (fully glycosylated). The expected canonical bonds based upon a theoretical digest with endolys-C are: 1:K1-1:K3, 1:K6-1:K12, 1:K13-2:K12, 2:K1-2:K5, 2:K7-2:K8, 2:K13-3:K1, 2:K14-2:K18 and 2:K24-2:K28 (associated peptide sequences provided below). Acceptance criteria were based upon a calculated average response ratio (ARR) between non-alkylated and alkylated peptides for each sample. Peptides with a MS response > 1E6 and a corresponding response ratio < 2x ARR were accepted. In the endoS2 treated samples two additional intra-chain disulfide bonds met these acceptance criteria; 1:K12-1:K13 (intra-light chain bond), 2:K1-2:K5 (intra-heavy chain bond). *For several peptides in the deglycosylated samples (across all lots) the MS responses were lower than expected, most likely due to solubility issues with the deglycosylated protein. As a result bonds 1:K1-1:K3 and 2:K1-2:K5 were found below the 1E6 threshold, however met the remaining criteria. Overall there was no lot-to-lot variation across all assays.*

DS bond	Fully Glycosylated			EndoS2 Treated			Deglycosylated		
	Lot A	Lot B	Lot C	Lot A	Lot B	Lot C	Lot A	Lot B	Lot C
1:K1-1:K3	🏱	🏱	🏱	🏱	🏱	🏱	🏱*	🏱*	🏱*
1:K6-1:K12	🏱	🏱	🏱	🏱	🏱	🏱	🏱	🏱	🏱
1:K13-2:K12	🏱	🏱	🏱	🏱	🏱	🏱	🏱	🏱	🏱
2:K1-2:K5	🏱	🏱	🏱	🏱	🏱	🏱	🏱*	🏱*	🏱*
2:K7-2:K8	🏱	🏱	🏱	🏱	🏱	🏱	🏱	🏱	🏱
2:K13-3:K1	🏱	🏱	🏱	🏱	🏱	🏱	🏱	🏱	🏱
2:K14-2:K18	🏱	🏱	🏱	🏱	🏱	🏱	🏱	🏱	🏱
2:K24-2:K28	🏱	🏱	🏱	🏱	🏱	🏱	🏱	🏱	🏱
1:K12-1:K13	🏳	🏳	🏳	🏱	🏱	🏱	🏳	🏳	🏳
2K8-2:K12	🏳	🏳	🏳	🏱	🏱	🏱	🏳	🏳	🏳

Identified peptide sequences [chain (L or H) shown in bold]:

1:K1-1:K3 (L23:L88)
DIQMTQSPSSLSASVGDRVTITCRASQDVNTAVAWYQQKPGK=LLIYSASFLYSGVPSRFSGSRSGTDFTLTISSLQPEDFATYYC QQHYTTPPTFGQGTK;
1:K6-1:K12 (L134:L194) SGTASVVCLLNNFYPREAK=VYACEVTHQGLSSPVTK;
1:K13-2:K12 (L214:H223) SFNRGEC=SCDK;
2:K1-2:K5 (H22:H96)
EVQLVESGGGLVQPGGSLRLSCAASGFNIK=NTAYLQMNSLRAEDTAVYYCSRWGGDGFYAMDYWGQGTLVTVSSASTK;
2:K7-2:K8 (H147:H203)
STSGGTAALGCLVK=DYFPEPVTVSWNSGALTSGVHTFPAVLQSSGLYSLSSVVTVPSSSLGTQTYICNVNHKPSNTK;
2:K13-3:K1 (H229:H232) THTCPPCPAPELLGGPSVFLFPPKPK=THTCPPCPAPELLGGPSVFLFPPK;
2:K14-2:K18 (H264:H324) DTLMISRTPEVTCVVVDVSHEDPEVK=CK;
2:K24-2:K28 (H370:H428) NQVSLTCLVK=SRWQQGNVFSCSVMHEALHNHYTQK

1:K12-1:K13 (L194:L214) VYACEVTHQGLSSPVTK=SFNRGEC;
2:K8-2:K12 (H203:H223)
DYFPEPVTVSWNSGALTSGVHTFPAVLQSSGLYSLSSVVTVPSSSLGTQTYICNVNHKPSNTK=SCDK

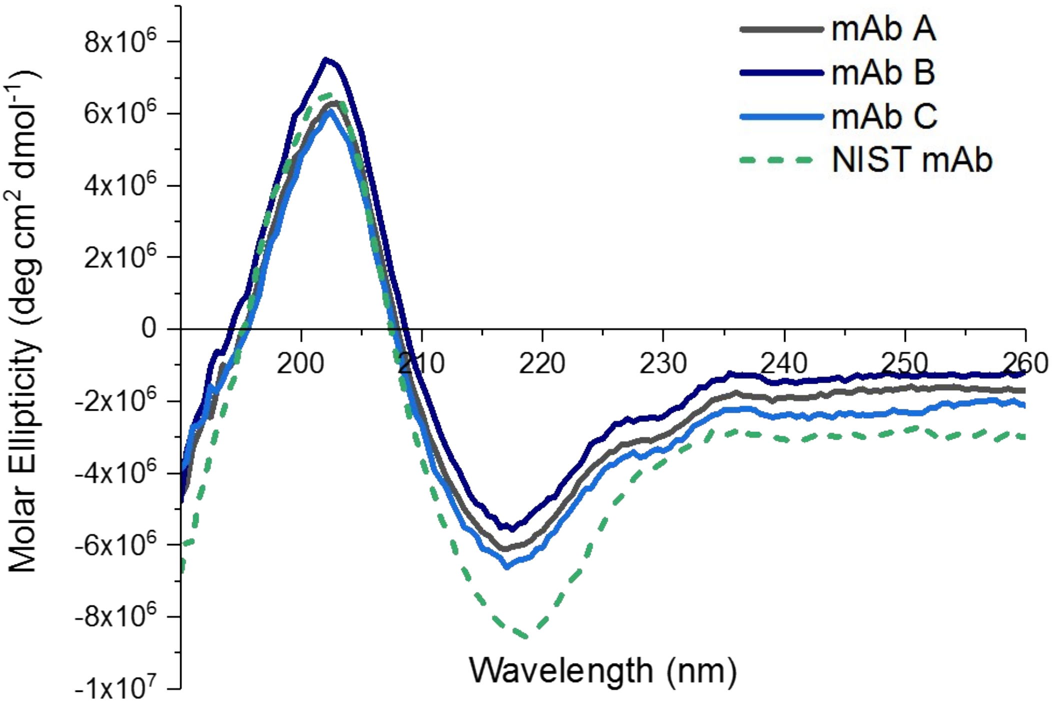

Figure 23: CD spectra for the three Herceptin lots (mAb A, B and C) alongside the CD spectrum for the NIST mAb for comparison to an IgG1 standard.
